# Combined diet and physical activity effects on health-related outcomes in people with overweight or obesity: an overview of systematic reviews

**DOI:** 10.3389/fnut.2026.1821389

**Published:** 2026-05-13

**Authors:** Atef Salem, Achraf Ammar, Khaled Trabelsi, Omar Boukhris, Juliane Heydenreich, Hadeel Ali Ghazzawi, Adam Tawfiq Amawi, Giuseppe Grosso, Piotr Zmijewski, Haitham Jahrami, Waqar Husain, Hamdi Chtourou, Wolfgang I. Schöllhorn

**Affiliations:** 1 Department of Training and Movement Science, Institute of Sport Science, Johannes Gutenberg-University Mainz, Mainz, Germany; 2 High Institute of Sport and Physical Education of Sfax, University of Sfax, Sfax, Tunisia; 3 Research Laboratory, Molecular Bases of Human Pathology, LR19ES13, Faculty of Medicine of Sfax, University of Sfax, Sfax, Tunisia; 4 Department of Nutrition and Food Technology, School of Agriculture, The University of Jordan, Amman, Jordan; 5 Research Laboratory: Education, Motricity, Sport and Health, EM2S, LR19JS01, High Institute of Sport and Physical Education of Sfax, University of Sfax, Sfax, Tunisia; 6 Department of Movement Sciences and Sports Training, School of Sports Science, The University of Jordan, Amman, Jordan; 7 SIESTA Research Group, School of Allied Health, Human Services and Sport, La Trobe University, Melbourne, VIC, Australia; 8 Sport, Performance, and Nutrition Research Group, School of Allied Health, Human Services and Sport, La Trobe University, Melbourne, VIC, Australia; 9 Department of Experimental Sports Nutrition, Faculty of Sports Sciences, Leipzig University, Leipzig, Germany; 10 Department of Biomedical and Biotechnological Sciences, University of Catania, Catania, Italy; 11 Department of Biochemistry, Gdansk University of Physical Education and Sport, Gdańsk, Poland; 12 Government Hospitals, Manama, Bahrain; 13 Department of Psychiatry, College of Medicine and Health Sciences, Arabian Gulf University, Manama, Bahrain; 14 Department of Humanities, COMSATS University Islamabad, Islamabad, Pakistan; 15 Research Unit, Physical Activity, Sport, and Health, UR18JS01, National Observatory of Sport, Tunis, Tunisia

**Keywords:** bi-domain intervention, body composition, cardiometabolic outcomes, obesity/overweight, umbrella review

## Abstract

**Background:**

This overview synthesized evidence from systematic reviews (SRs) of combined physical activity (PA) and diet interventions versus diet-only or PA-only approaches on obesity-related anthropometric, cardiometabolic, and physical fitness outcomes, and identified program features linked to better effects.

**Methods:**

We conducted an Overview of SRs following Cochrane guidance and PRIOR standards. Five databases were searched until June 13, 2024, and Google Scholar was updated on January 25, 2025. Eligible SRs synthesized randomized controlled trials of combined PA and diet interventions in people with overweight or obesity. We extracted key intervention characteristics, outcomes, and meta-analytic estimates where available. Methodological quality was assessed with AMSTAR 2, and overlap was quantified using the corrected covered area (CCA).

**Results:**

Thirty-two SRs (19 meta-analyses) met inclusion criteria. In adults, combined PA and diet interventions generally outperformed single-component comparators for reducing weight, BMI, waist circumference, and fat mass, and improving cardiorespiratory fitness. More favorable and sustained effects were most often reported in programs lasting ≥6–12 months with frequent, structured contact and supervised, resistance-inclusive exercise. In children and adolescents, face-to-face and family-engaged programs produced the most consistent improvements in BMI/BMI-z, but effects often attenuated 6–12 months after program end without maintenance. In older adults, combined programs reduce fat mass while preserving lean mass, strength, and function. Across diverse settings (including type 2 diabetes, workplaces, and digital delivery), combined interventions improved glucose control, insulin resistance, lipids, and inflammation, and some benefits occurred even without additional weight loss. Long-term lifestyle programs reduced incident type 2 diabetes by about half. Review quality varied (31% high; 69% low/critically low) and overlap between SRs was minimal (CCA 0.0–0.6%).

**Conclusion:**

Combined PA and diet interventions tended to outperform diet-only and PA-only approaches. The most consistent and durable benefits were linked to longer duration, higher contact, supervision, resistance training, and family engagement in youth, underscoring maintenance planning. These findings support prioritizing structured lifestyle programs, including scalable hybrid/digital delivery models, while interpreting effects cautiously given the predominance of low-quality SRs. Future research should improve rigor, standardize outcomes, report intervention dose and behavior-change methods, include longer follow-up, and improve representation of underserved groups.

## 1 Introduction

Obesity has emerged as a major global public health crisis. By 2022, its burden encompassed more than one billion individuals, about one in eight worldwide, and its prevalence has more than doubled among adults and quadrupled among young people since 1990 (1). However, temporal increases have not been uniform across high-income countries with stable food availability. For example, in the United States, measured adult obesity increased from 13.4% in 1960–1962 to 40.3% in August 2021–August 2023, whereas Japan has remained among the leanest OECD countries, underscoring the importance of differences in food environments, lifestyles, and policy contexts when interpreting global trends (2). Beyond its health consequences, the economic implications are equally profound: systematic reviews estimate that obesity already imposes multi-trillion-dollar annual costs on health-care systems and productivity worldwide (3). These figures underscore the urgency of scalable, evidence-based interventions that address positive energy balance while also acknowledging that obesity is shaped by interacting biological, behavioral, and environmental determinants, including appetite regulation, food quality and processing, physical activity, and the broader food environment. Even in settings with stable food availability, substantial cross-country variation suggests that obesogenic exposures and dietary patterns differ meaningfully across populations.

Obesity has been addressed through multiple strategies, most commonly dietary modification, increased physical activity (PA), and, often most effective, their combination. The World Health Organization Guidelines (2020) on PA and Sedentary Behavior recommend that adults accumulate 150–300 min week^−1^ of moderate-intensity activity (or the vigorous equivalent), emphasizing that “any activity is better than none” (4). Complementary WHO dietary guidance emphasizes not only limiting free sugars, sodium, saturated fat, and trans-fat, but also improving overall dietary quality by prioritizing whole grains, vegetables, fruits, and pulses, with adults encouraged to consume at least 400 g/day of fruits and vegetables and 25 g/day of naturally occurring dietary fiber (5, 6). Emerging evidence further suggests that obesity risk is shaped not only by excess energy intake in a general sense but also by low consumption of fiber-rich minimally processed foods and high exposure to ultra-processed dietary patterns, which may influence satiety, metabolizable energy, and downstream metabolic health (7–9). At the same time, cross-population doubly labeled water studies indicate that reduced daily energy expenditure alone is unlikely to fully explain rising obesity prevalence in industrialized settings, even though physical activity remains critically important for cardiometabolic health, body composition, and weight-loss maintenance (10). However, despite these clear prescriptions, population adherence remains low, prompting clinical and public-health guidelines, including the WHO Acceleration Plan to Stop Obesity (2023–2030), to endorse multicomponent lifestyle programs that integrate structured PA counseling with tailored dietary modification.

A growing evidence base underscores the added value of addressing both diet and PA. Consistent with this, a meta-analysis of randomized trials found that combined PA and diet intervention achieved greater weight loss than diet-only or exercise-only approaches at 12 months (11). A recent meta-analysis supports those findings; Rotunda, Rains (12) pooled short-term trials and reported greater weight loss versus usual care, with most interventions yielding clinically meaningful reductions for a sizeable proportion of participants. Evidence in younger populations is convergent; a meta-analysis showed significant reductions in body weight and metabolic-syndrome markers when combined PA and diet intervention were reinforced with behavior-change techniques (13). The available evidence further suggests that combined PA and diet intervention are associated with clinically meaningful weight loss and more favorable improvements in insulin sensitivity, lipid profiles, inflammation, and visceral fat compared with single-component approaches, with effects on visceral fat appearing more pronounced under dietary energy restriction (14). Evidence further suggests that specific pairings may matter. Resistance training combined with higher-protein, moderate-energy-deficit diets has been associated with better preservation of fat-free mass, whereas aerobic training is more consistently linked to greater fat loss (15). In adults with obesity and type 2 diabetes, combined PA and diet intervention are also associated with more favorable cardiometabolic profiles, even when weight loss is modest (15).

Nonetheless, important uncertainties remain that limit translation into practice and policy. Although numerous systematic reviews have examined combined PA and diet intervention, heterogeneity in intervention intensity, supervision, theoretical grounding, and delivery mode complicates comparison across reviews and challenges the consistency of guideline development. Outcomes are reported inconsistently, ranging from weight alone to waist circumference, glycemic indices, quality of life, and healthcare use, which further limits the ability of decision-makers to prioritize outcomes most relevant to specific populations and settings. Evidence on equity and scalability is limited, with few analyses by sex, age, or socioeconomic status. The therapeutic landscape has also changed rapidly with the emergence of highly effective incretin-based anti-obesity pharmacotherapies. In large contemporary trials, semaglutide 2.4 mg and tirzepatide have produced mean weight reductions substantially greater than those usually reported in older lifestyle-only trials (16, 17). At the same time, such therapies are used alongside, rather than instead of, reduced-calorie diets and increased physical activity. This broader context makes it important to interpret the present overview not only in terms of relative superiority over single-component lifestyle approaches, but also in relation to the absolute magnitude and clinical role of lifestyle-related benefits. Importantly, pharmacotherapy and lifestyle treatment should not be viewed as competing options; current obesity care increasingly combines anti-obesity medications with structured dietary and physical-activity support, while metabolic bariatric surgery remains the most effective durable treatment for severe obesity.

Given the rapid expansion of systematic reviews in this field, an overview of reviews is particularly valuable to synthesize evidence across the life course, identify intervention characteristics consistently associated with more favorable outcomes, and appraise the robustness of conclusions in light of review overlap and methodological quality. Such synthesis is especially relevant for clinicians designing lifestyle-based obesity services, public-health and workplace health programs, developers of digital and hybrid interventions, and pediatric and family-based care pathways, as well as for policymakers tasked with guideline development and resource allocation.

Accordingly, the present Overview of systematic reviews (OoSRs) aims to synthesize and critically appraise evidence on the effectiveness of combined PA and diet intervention on anthropometric, metabolic, physical-fitness, and patient-centered outcomes across the life course in populations with overweight or obesity.

## 2 Methods

The present OoSRs was conducted in accordance with the Cochrane Guide for Overviews of Reviews (18) and reported following the Preferred Reporting Items for Overviews of Systematic Reviews (PRIOR) Checklists (19).

### 2.1 Eligibility criteria

We prespecified eligibility using the PICOS framework (Participants, Interventions, Comparators, Outcomes, Study design). We included systematic reviews, with or without meta-analysis, which synthesized randomized controlled trials of combined PA and diet intervention in individuals living with overweight or obesity. To be considered a systematic review, publications were required to report, at minimum, a reproducible search strategy, explicit eligibility criteria, a systematic study selection process, and a structured synthesis of findings. Participants were children (≥ 5 years), adolescents, adults, or older adults classified as having overweight or obesity. Interventions were combined PA and diet intervention. Comparators were passive controls (usual care, wait-list), single-domain controls (diet-only or exercise-only), or attention controls. Outcomes had to include at least one post-intervention or follow-up measure related to anthropometry, metabolic health, patient-centered outcomes, or implementation and health-economic endpoints. We excluded reviews focused primarily on pharmacotherapy or bariatric surgery unless lifestyle-only arms were analyzed separately, reviews restricted to weight-neutral conditions (for example, sarcopenia), and narrative, scoping, or rapid reviews lacking systematic methodology. Only reviews published in peer-reviewed journals were included; non-English articles were translated prior to the study selection and data extraction phases. Cochrane reviews were eligible for inclusion. When multiple versions of the same systematic review were identified, the most recent and comprehensive version was retained.

### 2.2 Search strategy, study selection, and data extraction

The systematic literature search was conducted using five online databases (PubMed, Web of Science, CINAHL, ProQuest, and PsycInfo) from database inception to June 13th, 2024. The full research strategy and keywords were presented in Supplementary Table S1. References for all included papers were manually screened for additional relevant reviews. Google Scholar was searched on January 25th, 2025, for potentially published reviews.

The selection process and data extraction were conducted by two authors independently. After each step, the authors double-checked the spreadsheets’ accuracy, and any disagreements were solved by discussion between the two authors. Given the exploratory nature of the overview and the use of consensus resolution, formal inter-rater reliability statistics were not calculated. Duplicated articles were removed using the Endnote software (version 20). All articles were screened by the title, abstract, and full text using the PICOS inclusion criteria.

The following data were extracted and presented in Table 1: (i) reference, (ii) design, (iii) number of the included studies (N), (iv) participants characteristics, (v) intervention characteristics (PA. Diet, Delivery Mode, Duration, Comparator), (vi) measured outcomes (Outcome Domain, Specific Outcome, Follow-up), (vii) results, and (viii) conclusion.

**Table 1:** Summary of 32 systematic reviews included in this overview of systematic reviews.

Reference	Design	*N*	Participants	Intervention					Measured outcomes			Results	Conclusion
				PA	Diet	Delivery Mode	Duration	Comparator	Outcome Domain	Specific Outcome	Follow-up		
Aguilar Cordero, Ortegón Piñero (53)	SR	19	Children and adolescents with overweight/obesity. Individual study samples ranged from 24 to 1,047 participants; ages spanned ≈ 6–17 years (mixed sex)	Aerobic exercise, lifestyle physical activity, and/or strength work	Caloric-restricted or “healthy eating” counseling; some school-meal changes	Family-based, school-based, outpatient clinic or mixed settings; group and/or individual sessions	3 months to ≥2 years; authors emphasize ≥1 year as most effective	Usual care, minimal advice, or shorter/less intensive programs	Adiposity	BMI / BMI-z, body weight, % body-fat, waist circumference	3 months → 8 years (re-gain assessed ≥ 12 months)	Multicomponent programs lasting ≥ 1 year and involving family/school support produced durable BMI reductions.	Interventions combining PA + diet and lasting > 1 year, especially with family and school involvement, achieved sustained BMI reductions and lower cardiometabolic risk up to 3 years. Short-duration or stand-alone PA programs showed initial BMI loss but frequent rebound within a year Preserving lean mass (adding resistance work) and behavioral support reduced rebound risk. • Parental motivation strongly predicted adherence and weight maintenance
									Cardio-metabolic	Systolic and diastolic blood pressure, triglycerides, LDL-C, HDL-C, insulin resistance (HOMA-IR)	12 months → 3 years	In the Reinehr cohort, at 12 months children showed ↓ triglycerides 12%, ↓ LDL-C 5%, ↑ HDL-C 7%, ↓ HOMA-IR 17% and lower blood-pressure; all improvements were maintained through 3-year follow-up.	
									Quality of life	Child- and parent-reported HRQoL (e.g., Health-Related QoL scales)	Baseline → 12 months	Outpatient family training improved both child- and parent-reported HRQoL scores at 12 months, indicating psychosocial as well as physical benefit.	
									Endocrine / biomarkers	Leptin, TSH, free triiodothyronine (FT3)	During weight-loss and weight-regain phases up to 3 years	Leptin fell with weight loss and rose proportionally during regain, mirroring adiposity changes. TSH and FT3 levels tracked weight trajectories, with higher values predicting later regain	
Albornoz-Guerrero, Garcia (40)	SR	29 RCTs	Total *n* = 4,434; mean age 9.3 years; 56% girls; all classified as overweight/obese (BMI-based) at baseline.	Recommendations, structured exercise programs, or recreational games	Healthy eating recommendations, traffic light meal plans, cooking classes	Mostly group sessions in health-center or school settings; ~34% led by exercise professionals. Nutritionist-led (52% of studies)	≤ 6 months in 59% of studies; ≥ 12 months in 41%	Usual care / minimal advice / lower-intensity PA. Usual diet advice.	Anthropometric	BMI, BMI-z, BMI-SDS, % body-fat, waist circumference	Post-program (all studies); ≤ 6 months follow-up (43%); ≥ 12 months follow-up (57%)	BMI/BMI-z: 72% of the 29 interventions reported a statistically significant reduction immediately after treatment, when stratified by program length this rose to 77% for ≤ 6 months and 89% for ≥ 12 months Weight-loss maintenance: Of nine studies with extended follow-up, 77% maintained significant BMI improvements at ≥ 12 months.	Longer (≥ 12 months) family-centred PA + diet interventions sustain BMI improvements in children living in cold climates, while cardiometabolic and psychological benefits are less consistent.
									Physical / health condition	PA level, dietary intake, blood pressure, lipids and metabolic biomarkers, sedentary time, aerobic fitness	Same time-points as above	PA level increased in 48% of studies measuring it, typically via ≥ 10% rise in accelerometer-measured MVPA or self-reported activity scores. Dietary intake improved (e.g., ↑ fruit/veg, ↓ sugary drinks) in 41%; blood-pressure or lipid profiles improved in roughly one-third of studies, most often when interventions lasted ≥ 12 months and included structured exercise plus nutrition plans.	
									Psychological health	Health-related quality of life, self-esteem, mood, anxiety, parent-reported strengths/difficulties	Mostly immediately post-program; limited (≤12 months) follow-up in ~¼ of studies	Gains in health-related quality of life were reported in 17% of all included trials; self-esteem/mood benefits appeared in smaller single-study reports, generally paralleling weight reduction.	
AlMarzooqi and Nagy (54)	SR	22	Children 6–12 years (some studies included 5- and 13-y-olds; total sample sizes not pooled). Six studies explicitly involved parents.	Classroom PA breaks, after-school MVPA, family walking programs, policy-driven PE time increases.	Nutrition education, fruit/vegetable provision, cooking classes, beverage substitution policies.	Predominantly school-based (*n* = 13), with community, home and multi-setting approaches.	8 weeks to 3 years; most (*n* = 5) lasted one academic year.	Usual-practice or non-equivalent control schools/communities	Anthropometrics	BMI/BMI-z, waist circumference	Post-intervention to 3 years	APPLE (NZ) children recorded BMI-z 0.09 units lower at 1 years and 0.26 at 2 years, plus −1 cm waist; follow-up still showed −0.22–0.30 BMI-z and a 19% drop in overweight. *Be Active Eat Well* slowed weight gain by ≈1 kg and waist by 3 cm over 3 years. *Kids Living Fit* hospital program cut BMI by 0.6 units and waist by 0.7″ at 24 weeks. School policy trials (USA and Australia) and YMCA community program showed smaller but significant BMI decreases; e.g., 43% of YMCA participants achieved clinically-meaningful weight change.	Multi-component, school-centered programs that combine structured physical activity, dietary change and parent engagement deliver the clearest anthropometric benefits (e.g., ≥0.25 BMI-z reduction over 2 years). Single-component schemes can shift behavior (e.g., +47 min after-school MVPA or sharp fruit choice gains) but seldom move BMI alone. Consistent, theory-based measurements and longer follow-up are needed to enable future meta-analyses and publication-bias checks.
									Lifestyle behaviors	PA time, screen time, diet	Post-intervention to 2 years	Classroom PA program in China increased daily PA energy-expenditure and duration; MVPA rose while controls fell. APPLE pupils accumulated +26 min day^−1^ moderate-to-vigorous activity, and Project Energize schools reported higher activity and less sedentary time. After-school environment study logged ~47 min active recreation, with more MVPA during free play than organized sessions.	
										Diet	3 months to 3 years	Gold Medal Schools pupils drank fewer soft drinks and walked/biked more at 1 year. Wisconsin Fresh Fruit and Veg Program saw 40% of students choose fruit vs. 21% veg at school, and >55% willing to try fruit at home. CHOPPS (UK) reduced fizzy-drink intake, but BMI change was non-significant at 3 years.	
									Knowledge/attitudes, psychosocial	Rosenberg Self-Esteem Scale scores.	Post-intervention to 1 years	Healthy Buddies’ peer-led scheme improved health-knowledge and attitudes among 5-13-y pupils. Fit WIC boosted staff self-efficacy for obesity-prevention counseling after 1 year. Family-based Aboriginal program raised lifestyle knowledge and self-efficacy along with modest metabolic benefits	
Al-Mhanna, Rocha-Rodriguesc (15)	SR + MA	13 RCTs	Adults (2454) with T2DM + overweight/obesity (BMI ≥ 25 kg m^−2^); mixed sex; no upper-age limit	Supervised aerobic training (walking, cycling, classes) 3–5 sessions wk.^−1^, moderate-to-vigorous.	Caloric-restricted or macro-modified, delivered by dietitians.	Mainly outpatient clinics: some home follow-up.	8 weeks. – 24 months	Standard/usual care.	Anthropometrics and body composition	BMI, body weight, fat-mass, waist and hip circumference, waist-to-hip ratio	Post-program (8 weeks) → 24 months	BMI decreased significantly, SMD − 0.33 (95% CI − 0.50 to −0.16). Body weight also fell markedly, SMD − 2.69 (−4.85 to −0.54). Waist circumference dropped only when comorbidities were reported, SMD − 0.27 (−0.52 to −0.02), and showed no clear change without them, SMD − 6.68 (−14.67 to 1.31). Fat mass showed no convincing shift, SMD − 0.19 (−0.43 to 0.05). Hip circumference remained essentially unchanged, SMD − 0.11 (−0.34 to 0.13). Waist-to-hip ratio was unaffected, SMD − 0.06 (−0.72 to 0.60).	A 12-month (median) regimen that blends regular aerobic exercise with structured dietary guidance delivers clinically meaningful improvements in weight, central adiposity, blood pressure, lipid profile, glycaemic control and low-grade inflammation in adults living with both obesity and type 2 diabetes. Effects are strongest for adipokines (↑ adiponectin, ↓ leptin) and inflammatory markers (↓ IL-6, ↓ CRP) where precise pooled estimates are available. Evidence quality is low-to-moderate owing to small trial numbers and heterogeneity, but absence of overt publication bias for key lipid outcomes lends credibility to the findings.
									Blood pressure	Systolic BP, Diastolic BP	8 weeks → 12 months	Systolic blood pressure fell modestly, SMD − 0.16 (−0.30 to −0.01), and diastolic blood pressure decreased in parallel, SMD − 0.19 (−0.33 to −0.06).	
									Lipid metabolism	Total-C, HDL-C, LDL-C, Triglycerides	8 weeks → 24 months	Total cholesterol declined, SMD − 0.45 (−0.75 to −0.15), and triglycerides followed suit, SMD − 0.42 (−0.78 to −0.06), while HDL-C (SMD + 0.09, −0.20 to 0.37) and LDL-C (SMD − 0.17, −0.62 to 0.28) showed no clear effect.	
									Glucose metabolism	HbA1c, fasting blood glucose, fasting insulin	8 weeks → 24 months	Glycaemic control improved: HbA1c decreased SMD − 0.52 (−0.93 to −0.10), fasting blood glucose lowered SMD − 0.92 (−1.85 to 0.02), and fasting insulin fell SMD − 0.32 (−0.62 to −0.01).	
									Adipokines	Adiponectin, Leptin	12 weeks (single study)	Adipokines shifted favorably, with adiponectin rising by about +1.4 μg mL^−1^ SMD + 1.39 (+0.90 to +1.89) and leptin dropping roughly −7.3 ng mL^−1^ MD − 7.31; SMD − 1.39 (−1.89 to −0.90).	
									Inflammatory markers	IL-6, CRP, TNF-α	8 weeks → 24 weeks (≤ 2 trials each)	Inflammation markers improved: IL-6 fell by −0.55 pg. mL^−1^ MD − 0.55 (−0.92 to −0.18) and CRP by −0.46 mg L^−1^ SMD − 0.46 (−0.68 to −0.25), whereas TNF-*α* remained unchanged SMD − 0.55 (−1.56 to 0.46).	
Angawi and Gaissi (39)	SR	32 RCTs	Children and adolescents 2–19 years; sample sizes ranged from 146 to 18,423 per RCT	Extra curriculum or policy-driven PA (≥60 min MVPA/day, cognitive-behavioral PA lessons, playground or timetable changes)	Nutrition education, healthy-snack / sugary-drink restrictions, fruit and veg schemes, family meal planning	In-person classroom sessions, group workshops, home visits, recreation-center programs, environmental/policy change	5 weeks – 3 years interventions; follow-up 5 weeks – 7 years	Usual curriculum / no intervention / standard care	Anthropometry	BMI, BMI-z, WC, %-body-fat, skinfolds, obesity prevalence	5 weeks – 7 years	BMI / BMI-z: ~⅓ of RCTs (7/32) showed meaningful drops; the multicomponent APPLE trial cut BMI-z 0.09 at 1 year and 0.26 at 2 years. Waist circumference: Few data; APPLE shrank WC ≈ 1 cm at 2 years and Be Active Eat Well slowed growth by ≈ 3 cm across 3 years; most other trials nil. Skinfolds: One long-term Swiss PA study found no change in triceps/subscapular folds. Body-fat %: Four trials; effects small or mixed; no consistent reduction. Overweight / obesity prevalence: Nine trials measured prevalence; APPLE cut overweight risk by 19% at 2 years, whereas large cluster studies showed mixed or null shifts.	There is reasonable evidence that multicomponent school programs combining structured PA time, nutrition education and a supporting home environment reduce childhood obesity; effectiveness in non-school settings remains uncertain. The authors call for theory-based RCTs in preschools, communities and especially in developing countries, plus longer follow-up to confirm sustainability
									Behavior	MVPA minutes, screen-time, fruit-and-veg intake	intervention end – 2 years	MVPA: Preschool PA curricula pushed children above the 60-min/day guideline; APPLE added +26 min/day at 2 years; U. S. after-school free-play yielded ≈ 47 min active time. Screen time / sedentary: Australia’s Switch-Play cut sedentary time alongside BMI; a U. S. preschool policy capped screen exposure (<30 min wk.^−1^) but did not quantify change. Fruit and vegetable intake: Wisconsin FFVP raised fruit selection to 40% of pupils and willingness at home (> 55%); a preschool policy trial also boosted intake. Sugary-drink intake: CHOPPS reduced fizzy-drink consumption, and APPLE discouraged sugary beverages (amount not reported).	
Appuhamy, Kebreab (58)	SR + MA	34	4,160 adults; 60% were overweight/obese at baseline; mean age ≈ 49 years, mean BMI ≈ 30 kg m^−2^.	Moderate-intensity aerobic exercise (brisk walking, cycling, jogging, swimming) ~ 40 min × 4 sessions wk.^−1^; one-third also added resistance training	Caloric-restricted plans (≈ 500 kcal d^−1^ deficit) with ≤ 30% total fat and ≤ 10% saturated fat; some promoted higher protein or fiber.	Face-to-face counseling in community, workplace or clinic settings, is often reinforced by printed material.	2–60 months (mean follow-up 14 months).	Usual diet and habitual activity (no structured counseling).	Anthropometry	Body-mass index (BMI)	2–60 months	BMI: Pooled mean difference (MD) – 1.61 kg m^−2^ (95% CI – 1.87 to – 1.35; *p* < 0.001, *I*^2^ 99.8%). Extra 100 kcal d^−1^ restriction predicted a further 0.22 kg m^−2^ loss.	A year of moderate aerobic exercise combined with a ~ 270 kcal/day low-fat diet modestly but significantly lowers insulin, glucose, blood pressure, triglycerides and BMI (HDL unchanged), with greatest gains in metabolically at-risk participants; supporting diet-exercise counseling for diabetes prevention.
									Blood pressure	Systolic BP (SBP)		SBP: MD – 2.77 mm Hg (− 3.87 to – 1.65; p < 0.001, *I*^2^ 99.4%). Adults with IGT/MetS benefited an additional – 3.23 mm Hg.	
									Glucose metabolism	Fasting glucose (FG)		Fasting glucose: MD – 0.18 mmol L^−1^ (− 0.26 to – 0.10; p < 0.001, I^2^ 99.4%). Follow-up without active counseling attenuated the gain.	
									Insulin dynamics	Fasting insulin (FI)		Fasting insulin: MD – 2.56 mU L^−1^ (− 3.70 to – 1.42; p < 0.001, I^2^ 99.5%). Greater calorie cuts strengthened the drop; adding resistance training blunted it.	
									Lipid metabolism	Triacylglycerides (TAG); High-density lipoprotein (HDL)		TAG: MD – 0.26 mmol L^−1^ (− 0.33 to – 0.19; *p* < 0.001, *I*^2^ 99.8%). Larger baseline TAG and tighter calorie limits amplified improvement. HDL: MD – 0.02 mmol L^−1^ (− 0.04 to + 0.01; *p* = 0.31); no significant.	
Baillot, Romain (44)	SR + MA	56	4,178 class II–III obese adults, mean age ≈ 43–50 years, ~75% women	Supervised endurance or mixed training 3–5 sessions week^−1^, or targeted step goals.	Dietary caloric restriction (≈ 500–700 kcal d^−1^ deficit)	–	Median follow-up 6 months (range 2 weeks – 61 months)	Usual care	Anthropometry	Body weight, BMI, waist-circumference, fat-mass	2 weeks – 61 months	Weight −8.9 kg (−10.2, −7.7) BMI − 2.8 kg m^−2^ (−3.4, −2.2) Waist −6.9 cm- Overall, with larger weight loss in interventions lasting > 12 months (−11.3 kg vs. − 7.2 kg short term). Fat-mass also fell significantly.	In adults with class II–III obesity, multicomponent lifestyle programs that include regular physical activity yield moderate yet clinically meaningful reductions in body weight, central adiposity, blood pressure, atherogenic lipids and fasting insulin, even without surgery. Longer and more frequent contact enhances weight loss, but HDL-C and fasting glucose respond inconsistently. High-quality RCTs with behavior and quality-of-life endpoints are still needed.
									Blood pressure	Systolic BP (SBP), Diastolic BP (DBP)	2 weeks – 61 months	DBP dropped 4–6 mm Hg and SBP fell significantly across pooled studies, indicating modest cardiovascular risk reduction.	
									Lipids	Total-cholesterol, LDL-C, HDL-C, triacylglycerides (TAG)	2 weeks – 61 months	Total-C, LDL-C and TAG decreased (e.g., Total-C − 0.99 mmol L^−1^ short term), while HDL-C showed no consistent change.	
									Glucose / insulin	Fasting glucose, fasting insulin	2 weeks – 61 months	Short programs cut fasting glucose by −0.53 mmol L^−1^; longer programs achieved a sizeable fasting-insulin fall (−34.8 mU L^−1^), supporting improved insulin sensitivity.	
									Behavior	Physical activity (steps, min wk.^−1^), dietary pattern scores	3 mo – 24 months (behavioral sub-set)	Nine studies showed ↑ physical activity (steps, mins/week); five showed healthier diet patterns; two RCTs reported no behavioral change vs. control.	
									Quality of life	SF-36 physical-function, pain, general-health, sleep	≤ 6 months post-program (2 studies)	Two moderate-quality studies found improvements in physical-function, pain, general health and sleep; gains partly faded by 6 months follow-up.	
Barte, Veldwijk (55)	SR + MA	22	Healthy adults with mean BMI 25–40 kg m^−2^.	All programs prescribed regular, supervised or home-based aerobic / mixed exercise as part of a structured weight-loss plan.	Caloric-restricted diet	Face-to-face counseling by healthcare professionals, at least five sessions per program	Interventions lasted ≥12 months (weight assessed at 1 years)	Each analysis compared BMI data within the same intervention; no external control arm was needed.	Anthropometry	Absolute weight change (kg); % weight change	12 months	Weight (kg): At 1 years, overweight participants lost 1.1 kg less than class-I obese and 1.5 kg less than class-II obese (*p* < 0.01 for both contrasts). Percentage weight change: No significant differences among the three BMI classes. Overweight vs. class-I obesity: −1.1 kg (favoring heavier class), *p* < 0.01. Overweight vs. class-II obesity: −1.5 kg, p < 0.01.	Across professionally guided diet-plus-exercise programs, 1-year weight loss differs only marginally between BMI 25–40 kg m^−2^ classes. Lifestyle interventions are therefore equally appropriate for adults who are overweight or have class-I/II obesity.
Batsis, Gill (41)	SR	19	Community-dwelling adults ≥ 60 years (mean 67–71 years) with BMI ≥ 30 kg m^−2^ (or abdominal obesity by WC); many were sedentary, frail or had functional impairment at baseline. Loss-to-follow-up ranged 0–13%	Structured aerobic (treadmill/ walking), resistance or mixed exercise, 3 × wk.^−1^, 60–90 min sessions; five trials used progressive resistance to preserve muscle mass.	Energy-restricted diets (−500 to −1,000 kcal d^−1^, ≥ 1 g protein kg^−1^ d^−1^) plus calcium ± vitamin D supplementation in two trials.	Face-to-face group classes and supervised exercise in research gyms; monthly to weekly dietitian visits.	6 to 18 months.	Usual care, weight-stable healthy-diet advice, or exercise-only/diet-only arms within the same study.	Anthropometry	Body weight, BMI, waist circumference	6–18 months	Anthropometry – Intervention arms lost 0.5–10.7 kg (0.1–9.3% body weight); weight loss was greatest when a dietary deficit was combined with exercise, and minimal with exercise alone.	In adults ≥ 65 years with obesity, diet-induced weight loss paired with supervised exercise yields the greatest improvements in weight, physical performance and quality of life, while preserving muscle and bone. Exercise alone boosts function but rarely reduces weight, diet alone risks sarcopenia. High-quality, longer RCTs in primary-care settings are still needed to guide geriatric obesity management.
									Physical function	VO₂ peak, 6-min walk, muscle strength, timed chair stands, Physical Performance Test	6–18 months	Exercise-only groups improved performance without weight loss (e.g., VO₂ peak ↑ 1.4 mL kg^−1^ min^−1^; muscle strength ↑ 174 → 190 lb. one-rep-max). Diet-plus-exercise produced the largest gains (VO₂ peak ↑ 3.1; chair-rise and 6-MWT all improved) and mitigated diet-induced losses of lean mass/bone.	
									Quality of life	SF-36 physical component, WOMAC pain/stiffness	6–18 months	Combined diet + exercise arms increased SF-36 physical composite by ≈ 8–15%, whereas diet-only produced modest (+3%) and control no change.	
									Body composition	Fat mass, fat-free mass, DEXA lean mass	6–18 months	Diet alone reduced fat mass but also fat-free mass; adding resistance exercise preserved lean tissue. Diet-only arms showed small but significant bone-density loss; exercise blunted this effect.	
									Metabolic and other	Insulin resistance (HOMA-IR), glucose, bone mineral density, inflammatory markers	6–18 months	Combined arms improved insulin sensitivity and inflammatory profiles more than exercise-only; adverse events were minor (occasional falls, transient musculoskeletal pain).	
Best, Avenell (42)	SR + MA	40	Infertile women and men (mean age 25–35 years) with BMI ≥ 25 kg m^−2^.	Supervised or advised aerobic sessions; some added resistance work (3–5 × week).	Hypo-caloric (1000–2000 kcal d^−1^) or macronutrient-modified diets; three very-low-calorie and one ketogenic diet	Mostly individual or small-group counseling; five programs partly group-based, two involved both partners.	6 weeks → 18 months (median ~6 months).	Usual care, waiting-list, or alternative weight-loss modality (e.g., metformin, orlistat).	Anthropometry	Weight, BMI, waist circumference	6 weeks – 18 months	Pooled weight change across RCTs −3.98 kg (CI − 4.85 to −3.12) for diet + exercise; best individual losses ~10 kg.	Non-surgical weight-loss strategies; especially combining calorie-restricted diet and aerobic exercise; enhance pregnancy odds and improve ovulation in overweight or obese infertility patients.
Bondyra-Wiśniewska, Myszkowska-Ryciak (64)	SR	18	Children and adolescents (n = 1,578) aged 6–18 years with overweight or obesity	In 83% of programs, typically structured daily exercise prescriptions or PA counseling.	Every program except one included calorie-controlled or macronutrient-guided dietary advice delivered by a dietitian/nutritionist.	Multi-disciplinary (dietitian/nutritionist plus physician in most cases, sometimes exercise specialist); many incorporated parental sessions.	<6 months (short) or ≥6 months (long); longer programs were more often associated with BMI reduction	Predominantly pre−/post designs; a minority used usual-care or standard-intervention controls.	Anthropometry	BMI, BMI-z, occasional waist circumference and body-fat metrics (all 18 studies).	Follow-up 4 weeks to 12 months post-program.	8 of 23 interventions achieved a statistically significant fall in BMI and/or BMI-z; success correlated with combined diet + PA content, dietitian and physician involvement, and ≥ 6-month duration.	Combined diet-plus-exercise programs run for 6 months or more, and delivered by a multidisciplinary team, consistently reduce BMI and, in tandem, improve lipids and blood pressure in overweight or obese children and adolescents.
									Cardiometabolic	total-, LDL-, HDL-cholesterol, triglycerides, systolic/diastolic blood pressure		Among the 14 interventions reporting lipids/BP, decreases were frequent for LDL-C (10 programs), total-cholesterol (9), triglycerides (5) and increases for HDL-C (5). Seven also reduced systolic and/or diastolic BP. Programs that cut BMI were the same ones that most often improved TC, TG, LDL-C and BP, whereas unchanged BMI aligned with unchanged BP.	
Brown, Smith (43)	SR + MA	29	Children and adults. Adult baseline BMI ranged 21–36 kg m^−2^	PA component – supervised aerobic or mixed sessions (school PE, community walking groups, men-only gym classes).	Calorie-restricted or culturally adapted healthy-eating advice; a few very-low-calorie or ketogenic diets.	Schools, community centers, workplaces, home visits; many used culturally tailored materials, sex-specific groups or bilingual facilitators.	3–48 months in adults (median ≈ 12 m); 3–24 m in children.	Usual routine, waiting list or alternative lifestyle program; some before–after studies had no external control.	Anthropometry	BMI or z-BMI, body weight, waist circumference	Children 3–24 m; Adults 3–48 m	Children – BMI/z-BMI: Network of five controlled school studies showed no between-group effect (SMD –0.01, 95% CI –0.29 to 0.28) and substantial heterogeneity; removing one outlier did not change the null finding. Children – Waist circumference: Also, non-significant (SMD –0.17, −0.37 to 0.04). Adults – Weight: Adjusted data from two RCTs favored intervention (MD –1.82 kg, −2.48 to −1.16). Unadjusted sensitivity analysis of three trials (after excluding one diabetic-care study) yielded MD –1.20 kg (−2.23 to −0.17). Adults – BMI and Waist: Pooled effects non-significant (BMI MD –0.25 kg m^−2^; WC MD –0.56 cm, both with high I^2^). Before–after evidence: 20 of 24 adult intervention arms reduced BMI from baseline (range −0.3 to −9.6 kg m^−2^); greater losses occurred in lower-quality or higher-BMI studies.	Evidence remains sparse and heterogeneous, but culturally-tailored diet-plus-activity programs can trim ~1–2 kg in South-Asian adults, whereas current school interventions show no clear anthropometric benefit for South-Asian children. More rigorous, longer RCTs; especially in young populations; are required.
Gea Cabrera, Caballero (34)	SR + MA	13 RCTs	Adult employees (30–60 years) with overweight/obesity and/or ≥1 metabolic-syndrome (MetS) risk-factor; settings largely office, healthcare, trucking and clergy workplaces.	Education on daily steps, structured exercise, or tele-monitored activity (present in 11/13 trials).	Personalized meal plans, calorie restriction, Mediterranean menus, or healthy-cafeteria lunches.	Mixed face-to-face plus web/apps/telephone in most trials; two were fully digital.	3–36 months (majority ≤12 months).	usual care/no intervention or delayed program	Adiposity	WC, BMI	≥3 months; most 6–12 months.	Lifestyle interventions reduced BMI by a mean −0.86 kg m^−2^ (95% CI –1.20 to −0.51; P < 0.001) and WC by −2.06 cm (−2.98 to −1.13).	Workplace wellness programs yield modest BMI and BP reductions, plus small cholesterol gains. Effects vary significantly and are vulnerable to bias. Coaching + exercise components boost results; info-only approaches may backfire. Long-term effectiveness (>1 year) remains unclear.
									Lipids	TC, HDL-c, LDL-c, TG		TC fell −6.83 mg dL^−1^ (−10.01 to −3.66), while HDL-c rose +0.83 mg dL^−1^ (0.07 to 1.59) and LDL-c declined −6.20 mg dL^−1^ (−9.60 to −2.81); TG were lower by −12 mg dL^−1^ (−18.69 to −5.31).	
									Blood pressure	SBP, DBP		SBP decreased −3.39 mm Hg (−5.92 to −0.86) and DBP by −2.89 mm Hg (−3.93 to −1.84),	
									Glycaemia	FBG		FBG modestly reduced by −1.23 mg dL^−1^ (−2.01 to −0.45)	
Hassan, Head (33)	SR	17 RCTs	Adults (mean age ≈ 54 years, 70% women) with severe obesity (BMI ≥ 40 kg m^−2^, or ≥ 35 kg m^−2^ + comorbidity)	PA component – supervised or home-based aerobic / resistance sessions, step goals or facility access (present in 15/17 trials).	Energy-restricted menus, portion-controlled meal replacements, very-low-calorie diets, macronutrient-specific (low-CHO, low-fat, Mediterranean) plans.	Group education, individual counseling, tele-monitoring, app or phone support; intensity varied widely.	3–48 months (majority ≤ 12 months).	Standard / minimal care, wait-list or diet-only controls	Adiposity	Body-weight (kg), BMI, % weight change.	3–24 months for weight; up to 48 months for % weight	Weight: lifestyle arms shed 1.0–11.5 kg more than controls within 3–24 months. BMI fell 0.3–4.0 kg m^−2^ more than in control groups over the same period. Percentage body weight: relative loss was 1–6.5% greater with lifestyle programs after 6–48 months.	Multi-component diet-plus-exercise programs consistently produced additional weight losses of roughly 1–12 kg versus usual care, with modest concurrent improvements in glycaemic control and blood pressure; however, the evidence base is heterogeneous, at moderate-to-high risk of bias, and lacks formal assessment of publication bias.
									Glycaemia	HbA1c		Eight trials showed an extra 0.0–0.9 percentage-point drop versus control (follow-up 3–48 months).	
									Blood pressure	SBP/DBP		Nine studies reported an additional 1–8.6 mm Hg decrease for lifestyle participants.	
									Lipids	Total-C, TG		Total cholesterol: of seven trials, three recorded larger reductions with lifestyle change; mixed reporting units prevented a numeric range. Triglycerides: in nine studies, eight favored lifestyle arms, but heterogeneous units again precluded pooling.	
									Quality of life	--		Two studies measured it, with one showing a 1.0–9.8-point gain over control at 3–6 months.	
Johns, Hartmann-Boyce (11)	SR + MA	8 RCTs	Adults ≥ 18 years with BMI ≥ 25 kg m^−2^; mean age 32–70 years; ≈ 86% women; studies from US, Sweden, Belgium.	PA: supervised or prescribed moderate-to-vigorous aerobic activity (eg, brisk walking) 3–5 d wk.^−1^; some trials added resistance or vibration training.	Hypocaloric low-fat, balanced, or very-low-energy regimens; occasional meal replacements.	Mainly face-to-face group or mixed group/individual sessions led by dietitians, exercise physiologists, or psychologists.	Intensive weekly contact for ~3–6 months, then tapered; outcomes captured at 3–6 months and 12–18 months.	Diet-only BWMP or PA-only BWMP matched for contact where feasible	Anthropometry	Body-weight change (kg) at 3–6 months and at 12–18 months	At 3–6 months and 12–18 months	For weight: * Combined vs. diet-only, 3–6 months participants in programs pairing diet with physical activity lost 0.62 kg more than diet alone (MD –0.62 kg, 95% CI –1.67 to 0.44; *p* = 0.25; I^2^ = 0%; PI –1.95 to 0.71). * Combined vs. diet-only, 12 months the advantage rose to 1.72 kg (MD –1.72 kg, 95% CI –2.80 to −0.64; *p* = 0.002; I^2^ = 3%; PI –3.17 to −0.27). * Combined vs. PA-only, 3–6 months combined programs achieved 5.33 kg greater loss than PA-only (MD –5.33 kg, 95% CI –7.61 to −3.04; P < 0.001; I^2^ = 82%; PI –32.8 to 22.16). * Combined vs. PA-only, 12–18 months) the gap widened to 6.29 kg (MD –6.29 kg, 95% CI –7.33 to −5.25; *p* < 0.001; *I*^2^ = 9%; PI –8.32 to −4.26).	Behavioral weight-management programs that combine diet and PA counseling deliver the biggest payoff; about 5–6 kg more loss than exercise alone and roughly 2 kg more than diet alone after a year; making the dual-component approach the most effective single-setting option for clinically meaningful weight reduction.
									Behavioral (diet)	Change in reported energy intake (kcal day^−1^) at 3 months and 12 months (two trials)	at 12 months	Across two trials, reported energy-intake changes differed inconsistently between groups and could not be pooled quantitatively.	
									Behavioral (activity)	Change in PA indices at 12 months (step-count, VO₂max, 400-m walk)		Four studies found no consistent between-group difference in PA measures such as step count or VO₂max at 12 months, although one trial reported a larger step increase for the combined arm.	
Khalafi, Azali Alamdari (48)	SR + MA	50 RCTs	Adults ≥ 18 years with BMI ≥ 25 kg m^−2^ (range 24.9–44), mixed sex (8 male-only, 21 female-only, 21 mixed). Mean age spectrum 20.8–70 years.	PA component type: aerobic (24 trials), resistance (6), combined (7), interval/HIIT (4), football or mixed modes (remainder); typical dose 120–360 min wk.^−1^, moderate–vigorous intensity; supervised in 26 trials, unsupervised in 15, mixed in 9.	Hypocaloric plans (−300 to −1,000 kcal d^−1^), VLCD (600–800 kcal), alternate-day fasting, Mediterranean, DASH, high-protein, low-fat/low-carb; all aimed at weight loss.	Face-to-face individual/group counseling ± home-based exercise; some tele/unsupervised dietary guidance.	2 weeks to 12 months (median ≈ 16 weeks).	Identical diet program without structured exercise.	Anthropometry	Body-weight	≥2 weeks to 12 months	The extra exercise yielded a small, non-significant weight difference of −0.30 kg (−0.70 to 0.08; *p* = 0.12) compared with diet alone.	In adults with overweight/obesity, coupling structured exercise with a hypocaloric diet yields small but statistically significant extra improvements in fasting glucose (≈ − 1.5 mg dL^−1^) and insulin resistance (−0.33 SD) relative to diet alone, without materially increasing weight loss.
									Glycemic	Fasting plasma glucose		Adding exercise to a hypocaloric diet lowered fasting plasma glucose by −1.46 mg dL^−1^ (95% CI –1.97 to −0.94; p = 0.001).	
									Fasting insulin	--		The combined program produced a nonsignificant change of SMD –0.16 (−0.34 to 0.01; *p* = 0.07) relative to diet alone.	
									Insulin-resistance	HOMA-IR		Insulin sensitivity improved, with SMD –0.33 (−0.61 to −0.05; *p* = 0.01) favoring diet + exercise.	
Khalafi, Sakhaei (47)	SR + MA	47	Children, adolescents and adults aged 7–70 years with BMI ≥ 25 kg m^−2^ (mean BMI range 21–44); ~60% female.	Aerobic (*n* = 24), resistance (6), combined (7), HIIT (4), football/yoga/other (6); 120–360 min wk.^−1^, moderate-to-vigorous, supervised in ~½ of trials.	Hypocaloric plans (−300 to −1,000 kcal d^−1^), VLCD (600–800 kcal d^−1^), alternate-day fasting, Mediterranean/DASH/high-protein/low-carb.	Face-to-face individual or group counseling; many mixed supervised + home programs.	2 weeks to 18 months (median ≈ 16 weeks).	Exercise or diet only	Anthropometry	Body-weight	≥ 2 weeks to 18 months	Diet + exercise produced an extra of weight loss beyond exercise alone −5.59 kg (−7.26 to −3.93; p < 0.001) or diet alone −1.78 kg (−2.55 to −1.00; p < 0.001).	Adding exercise to a calorie-restricted diet yields about −5.6 kg extra weight loss plus larger leptin ↓ (SMD –0.34) and adiponectin ↑ (SMD + 0.37) versus exercise alone, but when diet is already in place it adds only ~ − 1.8 kg more weight loss and does not further shift either adipokine.
									Adipokines	Leptin and adiponectin		Diet + exercise lowered leptin an extra SMD –0.34 (95% CI –0.47 to −0.20; *p* = 0.001) beyond exercise alone, and SMD –0.13 (−0.26 to 0.00; *p* = 0.06) beyond diet alone. The combination raised adiponectin by SMD + 0.37 (0.12 to 0.63; *p* = 0.004) compared with exercise alone, and by SMD + 0.10 (−0.02 to 0.22; *p* = 0.11) compared with diet alone.	
Liang, Zhao (52)	MA	118 RCTs	Children/adolescents 6–18 years who were overweight or obese	Supervised or school/home-based aerobic, resistance or mixed PA; intensity usually moderate-to-vigorous.	Calorie-restricted, low-fat/low-carb, or balanced meal plans delivered alone or alongside PA.	FTF group/individual sessions or MH apps/web.	1–18 months (median ≈ 6 months).	Named control group (wait-list/usual lifestyle-education) or another active node (e.g., FTF-PA, FTF-DI).	Adiposity	BMI, BMI *z*-score, waist circumference, body-fat percentage	6 months	FTF-PA + DI vs. control: BMI ↓ 0.98 kg m^−2^ (CrI –1.19 to −0.77) ; BMI z ↓ 0.10 SD (−0.15 to −0.04) ; WC ↓ 1.49 cm (−1.97 to −1.00) ; BFP ↓ 0.98% (−1.41 to −0.64) • FTF-PA + DI vs. FTF-PA: additional BMI drop −0.69 kg m^−2^ (−1.04 to −0.36) ; WC further ↓ 1.12 cm (−1.93 to −0.29) • FTF-PA + DI vs. FTF-DI: BMI extra −0.75 kg m^−2^ (−1.10 to −0.41) Single-component PA or DI alone did not beat control for BMI (e.g., FTF-PA MD –0.29 kg m^−2^, CrI –0.57 to 0.00).	The face-to-face combination of structured physical activity and dietary restriction produces the largest and most consistent improvements in BMI (≈ 1 kg m^−2^), BMI z, waist circumference and body-fat when compared with usual care or with either component alone
Ling, Robbins (38)	SR	32 RCTs	13,654 preschoolers (median age ≈ 4.5 years; 54% boys) from 10 countries; 23 trials targeted the general population (prevention) and 6 trials enrolled children already overweight/obese or at risk (management).	Teacher- or coach-led aerobic play, daily 20–45 min sessions, extra PE classes, screen-time reduction.	Nutrition education, healthy-snack policies, parent cooking classes; some trials modified school menus.	Mainly school-based (16/23 prevention trials) with teacher training; five community-based and two home-based programs; management trials ran in outpatient/primary-care settings.	4 weeks–24 months (mean ≈ 8 months)	Usual curriculum/care, wait-list, or minimal health education.	Anthropometry	BMI, BMI-z/BMI-percentile, waist-circumference, sum of skinfolds, % body-fat	Post-program; 6-, 12- or 24-mo	8 of 23 (≈ 35%) achieved statistically significant improvements; typically BMI reductions of 0.3–1 kg m^−2^ or 1–2 cm smaller waist; three of these maintained effects at 6–24 months. • Management trials: 4 of 6 produced additional BMI/BMI-z declines (often 0.5–1.0 SD) versus controls, and 5 showed sustained benefits up to 12 months. Effective programs almost always combined both PA and nutrition elements and actively involved parents.	Among preschoolers, few prevention programs move the needle on BMI, whereas parent-centered management interventions in clinical/community settings more consistently trim body size; across both aims, coupling structured activity with nutrition education and engaging parents appears crucial, but the evidence remains heterogeneous and has not been formally assessed for publication bias
Liu, Hong (49)	SR + MA	23	Adults (18–75y) with overweight/obesity (mean BMI 28–38 kg m^−2^)	PA component: aerobic, resistance, combined, or HIIT exercise.	Energy-restricted (calorie-reduction) diet.	Mostly supervised, center-based sessions, 3–7 times week^−1^.	8–72 weeks.	Identical calorie restriction without the exercise component.	Inflammation.	CRP, IL-6, TNF-α.	8–72 weeks	CRP fell modestly when exercise was added to diet (SMD –0.16; 95% CI –0.29 to −0.03). IL-6 showed no meaningful change (SMD –0.04; −0.18 to 0.11). TNF-*α* likewise remained unchanged (SMD –0.14; −0.31 to 0.03).	Adding structured exercise to a calorie-restricted diet yields a small but significant reduction in CRP, while IL-6 and TNF-α are unaffected overall.
Mattos, Da Cunha (67)	SR	12	Adults (18–75 years) with overweight/obesity (baseline BMI 28–45 kg m^−2^); sample sizes 6–285; mixed sex (4 female-only, 1 male-only)	PA component type – moderate-intensity aerobic sessions added in 2 studies; one trial directly contrasted diet + exercise vs. diet. Diet component type –Delivery	Hypocaloric plans (500–1,500 kcal d^−1^ deficit), very-low-calorie diets (600–800 kcal d^−1^) or macronutrient-specific menus (high-protein; high-fiber; low-fat; high-fat).	Supervised center-based programs or structured home plans; ECG-based HRV recorded supine/day/night	11 days to 8 months (≥ 3 months in 8/12 studies).	Baseline within-group change; one study had diet vs. diet + exercise.	Time-domain HRV	RR-interval, SDNN, RMSSD, pNN50, HR	8 weeks–8 months	Time-domain HRV: Across the 12 trials, mean RR-interval lengthened in 4 of 7 studies, SDNN or RMSSD rose in 5 of 8, and resting heart rate fell in 6 of 10, all signaling stronger parasympathetic drive whenever programs lasted ≥ 3 months and achieved at least 5% weight loss.	Overall, the program generally enhanced heart-rate variability by lowering resting heart rate and LF/HF ratio while boosting vagal indices, provided it lasted at least three months; blood pressure and glycemic markers were largely unchanged; most lipids were unchanged except for lower triglycerides in three studies; leptin fell in most studies with one null finding; adiponectin decreased in three of four studies.
									Frequency-domain HRV	LF, HF, LF/HF		High-frequency (HF) power; another vagal marker; increased in 2 of 4 studies, while the sympatho-vagal LF/HF ratio dropped in 5 of 8, indicating a shift toward vagal dominance after clinically meaningful weight reduction.	
									Nonlinear HRV	SD1, SD2		Three trials reported Poincaré-plot indices; two showed larger SD1 and SD2 values (greater beat-to-beat variability) after weight-loss interventions, whereas one found no change, suggesting a possible but still uncertain non-linear HRV benefit.	
									Glycaemia	Glucose		Most studies reported no change in blood pressure. Glycemic markers were largely unchanged, with no significant effects on blood glucose, insulin, or HOMA-IR. Lipids were mostly unchanged, although three studies found lower triglycerides. Leptin decreased in most studies, with one null finding. Adiponectin decreased in three of four studies.	
									Blood pressure	SBP and DBP			
									Lipids	Lipid			
									Insulin-resistance	Insuline and HOMA-IR			
									Adipokines	Leptin and adiponectin			
McGovern, Johnson (36)	SR + MA	30	Children and adolescents 2–18 years classified as overweight /obese	Structured aerobic sessions, activity counseling or sedentary-time reduction (20 trials).	Hypocaloric, low-glycemic, low-carb, high-protein or meal-replacement plans (6 trials).	School, family, clinic or community programs; some involved parents	6 weeks – 12 months (most ≤ 6 months for outcomes).	Usual lifestyle/education, placebo or minimal contact control.	Adiposity	BMI (primary), % body-fat, fat-free mass.	≤6 months	Combined diet + PA programs delivered a small-to-moderate BMI reduction overall, especially when parents were involved and children were ≤ 8 years (g ≈ −0.35; parental subgroup g = −0.70).	Exercise alone reshapes body fat but scarcely affects BMI, diet alone is marginal, and small-to-moderate additional benefit emerges when diet and activity are combined
Merlotti, Morabito (50)	SR + MA	5	Adults (mean age ≈ 54 years) with BMI ≥ 30 kg m^−2^; ~70% had impaired fasting glucose and/or impaired glucose tolerance at baseline	Supervised or home-based aerobic / mixed exercise, ≥150 min wk.^−1^ at moderate intensity.	Energy-restricted or balanced diet targeting 5–10% weight loss.	Outpatient group or individual sessions led by dietitians/physiotherapists; boosters every 1–3 months.	1–15 years follow-up (median ≈ 13 years).	Usual care or minimal lifestyle advice.	Incident type 2 diabetes.	Number of new diabetes cases during follow-up.	1–15 years	Combined PA + diet programs reduced the risk of developing type 2 diabetes by ~56% versus control (OR 0.44, 95% CI: 0.36–0.52). Greater weight loss, younger age and higher baseline fasting-insulin predicted the largest risk reductions.	In adults with obesity, a long-term program that combines structured exercise with dietary calorie restriction roughly halves the likelihood of progressing to type 2 diabetes (OR ≈ 0.44) with minimal heterogeneity and no clear publication bias.
Miller, Fraser (46)	SR	14	Participants were adults with BMI ≥ 30 kg m^−2^; mean age across trials 37–75 years; mean baseline BMI 31–37 kg m^−2^.	Supervised aerobic (65–85% HRmax, 90–225 min wk.^−1^), resistance (2–3 sets × 8–12 reps, 65–85% 1-RM), or combined modes.	Energy-restricted menus creating 400–1,000 kcal d^−1^ deficit (all trials).	Outpatient/group or individual sessions led by exercise professionals and dietitians; no bariatric or drug arms.	3–12 months (most 3–6 months).	Identical dietary restriction without structured exercise.	Body composition	Total mass, fat mass, lean mass	3–12 months	Linear-mixed-model estimates showed extra fat-mass loss with aerobic (−17.8% from baseline) or resistance (−17.3%) training versus diet alone (−14.8%), and smaller lean-mass loss (−2.2% to −2.5% vs. − 3.6%).	When obese adults follow a calorie-restricted diet, adding structured exercise consistently improves fitness and strength and shifts weight-loss quality; yielding a few extra percentage points of fat loss while preserving lean tissue.
									Cardiorespiratory fitness	VO₂peak (absolute; relative to body or lean mass)		Exercise + diet raised VO₂peak in all nine aerobic arms and out-performed diet-only in 4 of 7 direct comparisons, with absolute gains of 7–27% (≈ 2–5 mL kg^−1^ min^−1^).	
									Muscle performance	1-RM strength (upper/lower), isometric leg strength		Every resistance or combined program increased isotonic 1-RM, whereas diet-only produced little or negative change; between-group differences ranged 10–30%.	
									Functional capacity	Physical-Performance Test, gait speed, balance (subset of trials)		In the only study reporting ADL capacity, the Physical-Performance Test improved 21% with exercise + diet vs. 12% with diet alone, with faster gait and better balance.	
Olateju, Opaleye-Enakhimion (45)	SR	4	Adults 25–70 years, BMI 30–42 kg m^−2^	≥ 175 min wk.^−1^ of mixed endurance + strength sessions (center-based or home-based with monitoring); intensity moderate-to-vigorous.	Calorie-restricted menus (−500 to −1,000 kcal d^−1^) delivered with meal-plans or portion-controlled products; one study used alternate-day fasting.	Face-to-face sessions, telephone/online support, meal replacements.	6 weeks to 24 months (median 6 months).	Diet-alone, exercise-alone or usual-care groups	Adiposity	Body-weight, BMI, body-fat mass, visceral-fat area	6–24 months	Portion-controlled diet + ≥ 175 min wk.^−1^ exercise delivered an extra −5% body-weight loss over 24 months versus usual care. Energy-deficit diet + combined strength + endurance training reduced total fat mass more than the same diet with strength- or endurance-only training. Programs pairing fasting or Mediterranean-style diets with supervised aerobic sessions showed 2–4 kg greater weight loss than diet alone at 6–12 months	Across the four qualifying studies, coupling a hypocaloric diet with ≥ 175 min wk.^−1^ of mixed endurance + strength exercise consistently produced clinically meaningful extra fat and weight reduction (≈ 5% body weight over 6–24 months) compared with diet, exercise, or usual care alone.
Peirson, Fitzpatrick-Lewis (37)	SR + MA	31 RCTs	Overweight/obese children and adolescents aged 2–18 years; mean baseline ages 7–16 years	Supervised aerobic/strength or mixed-activity sessions (school, clinic or home based).	Individual/family nutrition counseling or calorie-controlled diet plans.	Mix of family-based and individually focused programs, usually group sessions with some one-to-one elements	3–24 months (5/6 trials ≤ 12 months).	No-intervention, usual care, wait-list or minimal-contact advice	Anthropometry	Change in BMI or BMI z-score	≥ 6 months after baseline	Diet + PA produced the largest weight-reduction effect, with a pooled standardized mean difference (SMD) of −1.09 (95% CI –1.84 to −0.34) for BMI/BMI z-score.	Diet plus exercise programs give overweight kids a brief boost in weight control, but results vary widely and typically fade once the sessions stop. At postintervention, these programs reduced blood pressure, did not meaningfully change lipid profiles or fasting glucose, improved overall quality of life, and provided no postintervention data on multistage fitness test performance.
									Blood pressure	SBP and DBP		Systolic and diastolic blood pressure decreased after the Diet + PA intervention (SBP: MD − 3.42 mmHg, 95% CI − 6.65 to −0.29, 5 studies; DBP: MD − 3.39 mmHg, 95% CI − 5.17 to −1.60, 5 studies).	
									Lipids	Total cholesterol, LDL-C, HDL-C, and triglycerides		Lipid profile showed no significant changes in total cholesterol, LDL-C, HDL-C, triglycerides, or fasting glucose.	
									Glycaemia	Fasting glucose			
									Quality of life	--		Quality of life improved (SMD 2.10, 95% CI 0.60 to 3.60, 6 trials)	
Albert Perez, Poveda González (35)	SR	27 RCTs	Adults ≥ 18 years meeting ATP III, IDF or WHO/OMS metabolic-syndrome criteria; mix of men and women (30 –100% female depending on trial); mean ages 30–70 years; baseline BMI typically 30–35 kg/m^2^.	Mostly supervised aerobic or combined aerobic + resistance sessions 3–5 d·wk.^−1^; some trials used interval training, walking programs or hiking at altitude.	Hypocaloric Mediterranean, low-fat, low-carbohydrate, DASH or protein-adjusted plans with macronutrient targets and nutrition counseling.	Face-to-face (individual or group), telephone or web coaching; several added tele-monitoring.	0.75 months (3 weeks hiking) to 36 months (Mediterranean-diet + exercise in primary care); majority 3–6 months.	Usual care, diet-only, exercise-only or minimal-contact advice.	Anthropometry	Body weight, body-fat mass or %, BMI, waist circumference	At end of intervention, only a few trials reported maintenance beyond 6 months.	Combined diet + PA consistently produced clinically meaningful reductions, especially in waist circumference (−5 to −15 cm) and body weight (−3 to −12 kg). Magnitude depended more on total energy deficit and exercise structure than on macronutrient ratio. Review highlights that energy-restricted diets paired with regularly structured exercise yield the most favorable body-composition changes.	Energy-restricted dietary counseling plus structured physical activity reliably improves weight and, most of all, abdominal adiposity in adults with metabolic syndrome, but evidence is drawn from diverse short- to medium-term RCTs and lacks pooled estimates or bias assessment.
Rotunda, Rains (12)	SR + MA	14 RTCs	2,407 adults (mean age ≈ 40–52 years) with overweight/obesity (BMI ≥ 25); baseline weight 82–139 kg	Supervised aerobic or circuit sessions, step-count goals, motivational interviewing, self-monitoring	Hypocaloric or tailored calorie goals, nutrition education, meal plans/replacements	7 fully online/remote, 4 in-person, 3 mixed	8–26 weeks. (< 13 weeks = 7; 13–26 weeks = 7)	Wait-list, low-touch information, or usual care	Anthropometry	Mean change in body weight	8–26 weeks	Pooled mean extra loss MD = −2.59 (−3.47 to −1.72) versus controls; effects comparable for < 13 weeks. vs. 13–26 weeks. programs. Larger effects observed in fully online delivery and male-only trials.	Brief diet-plus-exercise programs consistently for a few months consistently beat usual care for weight loss, especially when run fully online.
Ruiz-González, Cavero-Redondo (56)	SR + MA	7 RCTs	Women (15–49 years) with overweight/obesity (baseline BMI 30–42 kg m^−2^)	Mostly moderate-intensity aerobic sessions (≥ 150 min wk.^−1^) with some resistance or interval training.	Hypocaloric healthy-eating plans (500–800 kcal deficit; 1,200–1,600 kcal d^−1^), occasionally high-protein (≈ 35% energy).	Supervised face-to-face classes plus home tasks; some added dietitian counseling.	4 weeks to 12 months (median 12 weeks).	Placebo/no-treatment, usual care or minimal dietary advice.	Anthropometry	BMI change		Diet + exercise produced moderate extra BMI loss and a small, MD = −1.42 kg m^−2^ (95% CI –1.76 to −1.09)	For women with overweight or obesity, pairing calorie-restricted eating with structured exercise trims BMI by about 1½ kg/m^2^ beyond usual care
Salam, Padhani (57)	SR + MA	56	Children and adolescents; majority school-aged; baseline BMI classified as healthy-weight to obese depending on prevention vs. management cohort	Structured exercise or PA promotion delivered in schools / communities (aerobic games, PE classes, walking programs).	Nutrition education and/or energy-restricted healthy-eating guidance.	Predominantly face-to-face group sessions in schools or community centers; some home-tasks.	≥12 weeks (range 3 months – 1 years; median ≈ 6 months).	Usual curriculum / no-intervention / minimal advice controls.	Anthropometry	BMI *z*-score, BMI, body weight	≥6 months	Diet + exercise interventions outperformed controls in prevention trials (BMI-z: −0.12 [−0.18 to −0.06]; weight: −1.59 kg [−2.95 to −0.23]) despite high heterogeneity. In management trials, they provided modest short-term weight loss (−2.07 kg [−2.90 to −1.24]).	School- or community-based programs that pair structured physical activity with nutrition education trim BMI modestly in children and teens, but effects are highly variable and evidence of lasting benefit or publication-bias assessment is still missing.
Schwingshackl, Dias (32)	SR + MA	22	Adults ≥ 19 years with BMI ≥ 25 kg m^−2^ (mean 25.6–38.2 kg m^−2^); ages 35–70 years	PA component – mostly supervised aerobic or combined aerobic + resistance sessions (50–85% HR_max), 2–5 d wk.^−1^.	Energy-restricted low-fat or balanced diets (≈ 500–1,000 kcal deficit; ≤ 30% energy from fat) or meal-replacement plans	Mix of face-to-face group or individual sessions, some partially home-based.	Interventions themselves ≥12 months; total follow-up ≤72 months.	Diet-only, exercise-only or usual-care/minimal-contact controls.	Anthropometry	Body weight	≥ 12 months	Diet + exercise trimmed an extra 1.38 kg beyond diet alone (95% CI –1.98 to −0.79; I^2^ 0%) and 4.13 kg beyond exercise alone (−5.62 to −2.64; I^2^ 77%).	Across year-long or longer trials, coupling energy-restricted eating with structured exercise systematically out-performs either strategy alone: the biggest and most reliable gains appear in body weight, abdominal fat, blood lipids and aerobic fitness, while improvements in blood pressure are modest. The added exercise component amplifies and sustains dietary benefits rather than merely duplicating them.
										Waist circumference		Abdominal girth fell a further 1.68 cm relative to diet (−2.66 to −0.70; *I*^2^ 0%) and 3.00 cm relative to exercise (−5.81 to −0.20; *I*^2^ 69%).	
										Fat mass		Combined programs removed an additional 1.65 kg of fat vs. diet (−2.81 to −0.49; I^2^ 61%) and 3.60 kg vs. exercise (−6.15 to −1.05; I^2^ 92%).	
										WHR		The dual approach produced a very small yet significant extra reduction of 0.01 units versus each single component.	
									Blood pressure	SBP; DBP		DBP; Falls of 1.2 mmHg vs. diet (−2.3 to −0.1; I^2^ 28%) and 2.1 mmHg vs. exercise (−3.4 to −0.7; I^2^ 0%). S BP; Minimal difference vs. diet, but ~2.8 mmHg lower than exercise (−4.5 to −1.1; I^2^ 0%).	
									Blood lipids	TC		No clear advantage over diet (−2 mg dL^−1^, CI includes 0) but ~11 mg dL^−1^ lower than exercise alone (−15.9 to −6.8; I^2^ 0%).	
										LDL-C		Difference negligible vs. diet, but ~10 mg dL^−1^ lower than exercise (−14.3 to −5.8; I^2^ 8%).	
										HDL-C		Slight rise of 1.6 mg dL^−1^ over diet (0.3 to 3.0; I^2^ 51%); no meaningful change vs. exercise.	
										TG		About 10 mg dL^−1^ lower than diet (−17.4 to −2.8; I^2^ 0%); effect vs. exercise uncertain (CI crosses 0).	
									Cardiorespiratory fitness	VO₂ max.		Fitness jumped by 3.6 mL kg^−1^ min^−1^ over diet (2.1 to 5.1; I^2^ 88%) and 2.1 mL kg^−1^ min^−1^ over exercise (1.5 to 2.7; I^2^ 9%).	
Selvendran, Penney (51)	SR + MA	9	Children aged 7.4–12.4 years, all had BMI ≥ 25 kg m^−2^ (overweight/obese).	PA component: supervised aerobic / game-based exercise sessions, usually weekly or twice-weekly.	Calorie-restricted or low-glycemic-load diet education plus behavioral skills (goal setting, self-monitoring).	7/10 trials family-based group programs; others delivered in schools or community clinics.	6–18 months (most 12 months).	Wait-list, usual care or single information session	Adiposity.	Change in BMI	6 and 12 months; one trial reported to 18 months.	Combined diet + PA programs produce modest but significant reductions in BMI over 6–12 months: 6 months: MD = −1.34 kg m^−2^ (95% CI –2.19 to −0.50) vs. control. 12 months: MD = −1.52 kg m^−2^ (95% CI –2.97 to −0.07).	Well-structured programs that blend regular, supervised physical activity with concrete dietary guidance (often family-based) trim about 1–1.5 kg m^−2^ off children’s BMI in the first 6–12 months; noticeably better than “just advice” controls.

1-RM, one-repetition maximum; 6-MWT, six-minute walk test; ADL, activities of daily living; APPLE, name of the “APPLE” school-based obesity-prevention trial/program (as cited in included studies); ATP III, Adult Treatment Panel III criteria; BFP, body-fat percentage; BMI, body mass index; BMI-SDS, BMI standard deviation score; BMI-z, BMI z-score; CHO, carbohydrate; CHOPPS, name of the “CHOPPS” school-based intervention (as cited in included studies); CI, confidence interval; CrI, credible interval; CRP, C-reactive protein; DASH, Dietary Approaches to Stop Hypertension; DBP, diastolic blood pressure; DEXA, dual-energy X-ray absorptiometry; DI, dietary intervention (diet-only node/arm); ECG, electrocardiogram; FBG, fasting blood glucose; FG, fasting glucose; FI, fasting insulin; FT3, free triiodothyronine; FTF, face-to-face; g (effect size), Hedges’ g; HbA1c, glycated hemoglobin; HDL-C (HDL-c), high-density lipoprotein cholesterol; HF, high-frequency (HRV component); HIIT, high-intensity interval training; HOMA-IR, Homeostatic Model Assessment of Insulin Resistance; HR, heart rate; HRmax (HR_max), maximal heart rate; HRQoL, health-related quality of life; HRV, heart-rate variability; IDF, International Diabetes Federation; IGT, impaired glucose tolerance; IL-6, interleukin-6; I^2^, Higgins’ I-squared heterogeneity statistic; LDL-C (LDL-c), low-density lipoprotein cholesterol; LF, low-frequency (HRV component); LF/HF, low- to high-frequency power ratio; MA, meta-analysis; MD, mean difference; MetS, metabolic syndrome; MH, mobile health; MVPA, moderate-to-vigorous physical activity; N, sample size; NZ, New Zealand; OMS, Organisation mondiale de la Santé (World Health Organization); OR, odds ratio; p, *p*-value; PA, physical activity; PE, physical education; PI, prediction interval; pNN50, % of successive NN (normal-to-normal) intervals differing by >50 ms; RCT, randomized controlled trial; RMSSD, root mean square of successive differences; RR, R–R interval; RTC, randomized controlled trial (as written in table); SBP, systolic blood pressure; SD, standard deviation; SD1, Poincaré-plot short-term variability index; SD2, Poincaré-plot long-term variability index; SDNN, standard deviation of NN intervals; SDS, standard deviation score; SF-36, 36-Item Short Form Health Survey; SMD, standardized mean difference; SR, systematic review; T2DM, type 2 diabetes mellitus; TAG, triacylglycerides; TC, total cholesterol; TG, triglycerides; TNF-α, tumor necrosis factor-alpha; TSH, thyroid-stimulating hormone; UK, United Kingdom; USA, United States of America; VLCD, very-low-calorie diet; VO₂max (VO₂ max), maximal oxygen uptake; VO₂peak (VO₂ peak), peak oxygen uptake; WC, waist circumference; WHO, World Health Organization; WHR, waist-to-hip ratio; WIC, Special Supplemental Nutrition Program for Women, Infants, and Children; WOMAC, Western Ontario and McMaster Universities Osteoarthritis Index.

### 2.3 Methodological quality assessment

The methodological quality of the SRs was assessed using the AMSTAR2 (A MeaSurement Tool to Assess Systematic Reviews) (20) with 16-items. Seven items are considered critical, and three items concern meta-analytical methods and are not applicable for SRs without accompanying meta-analysis (Supplementary Table S4). The AMSTAR2 rates SRs as critically low (more than one critical weakness, with or without noncritical weaknesses), low (one critical weakness, with or without noncritical weaknesses), moderate (no critical weakness, with more than one non-critical weakness), or high quality (neither critical nor non-critical weaknesses) (20). AMSTAR2 was conducted independently by two authors, with any disagreements solved by discussion between the two authors. AMSTAR 2 was used to assess methodological quality at the review level; no separate tool for risk of bias across reviews [e.g., ROBIS (21)] was applied.

AMSTAR 2 was selected because it is designed to appraise the methodological quality of systematic reviews at the review level. By contrast, GRADE is primarily intended to rate the certainty of evidence for specific outcomes, and its application in overviews of reviews remains methodologically challenging and lacks consistent guidance, particularly when included reviews vary in scope, quality, outcome definitions, and overlap of primary studies (22–24). Therefore, formal GRADE assessments were not performed in the present overview. To mitigate the risk of overinterpretation, conclusions were instead contextualized by explicitly considering methodological quality (AMSTAR 2), consistency of findings across reviews, and the degree of overlap between primary studies.

### 2.4 Overlap of included studies

The degree of overlap was quantified using the corrected covered area (CCA) method (25). The CCA is calculated by dividing the frequency of repeated occurrences of index studies (first occurrence of a primary study) in other reviews (of the same domain) by the product of the number of index studies and the number of reviews, minus the number of reviews. The CCA is represented as a percentage between 0 and 100%. A CCA of 0–5% is considered a slight overlap, 6–10% moderate overlap, 11–15% high overlap, and more than 15% very high overlap (25). To visualize pairs of overlapping SRs in each domain, a heatmap was generated using the “ccaR” package in the R programming language^1^ (26, 27). In addition to quantifying overlapping, explicit decision rules were applied when interpreting findings from overlapping reviews. Priority was given to systematic reviews with higher AMSTAR 2 ratings, more recent search dates, and broader primary study coverage, to minimize double counting and undue influence of methodologically weaker reviews.

### 2.5 Data synthesis

Data synthesis followed best-practice recommendations for OoSRs. Where included reviews conducted meta-analyses, the pooled number of participants, pooled effect estimates (e.g., mean difference or standardized mean difference with 95% confidence intervals), heterogeneity statistics (*I*^2^), and model specifications were extracted as reported by the review authors. No re-analysis of primary study data was undertaken.

For reviews without meta-analysis, findings were synthesized narratively using a structured approach based on: (i) the direction of effects reported by the systematic reviews, (ii) consistency of findings across primary studies and reviews, (iii) clinical relevance of the reported effects, and (iv) the methodological quality of each review as assessed by AMSTAR 2. Narrative-synthesis judgments were made by two authors independently and resolved by consensus using these predefined criteria, although formal inter-rater reliability statistics were not calculated. When overlapping reviews addressed similar populations and outcomes, greater interpretive weight was given to reviews with higher methodological quality, more recent search coverage, and broader primary-study inclusion. Vote counting based solely on statistical significance was avoided, as this approach may bias interpretation by disregarding study size, precision, and between-study heterogeneity (28, 29).

When multiple systematic reviews addressed overlapping populations and outcomes, interpretation was guided by a structured hierarchy prioritizing higher methodological quality, more recent search coverage, and greater comprehensiveness of included trials.

## 3 Results

### 3.1 Study selection

**Figure 1:**
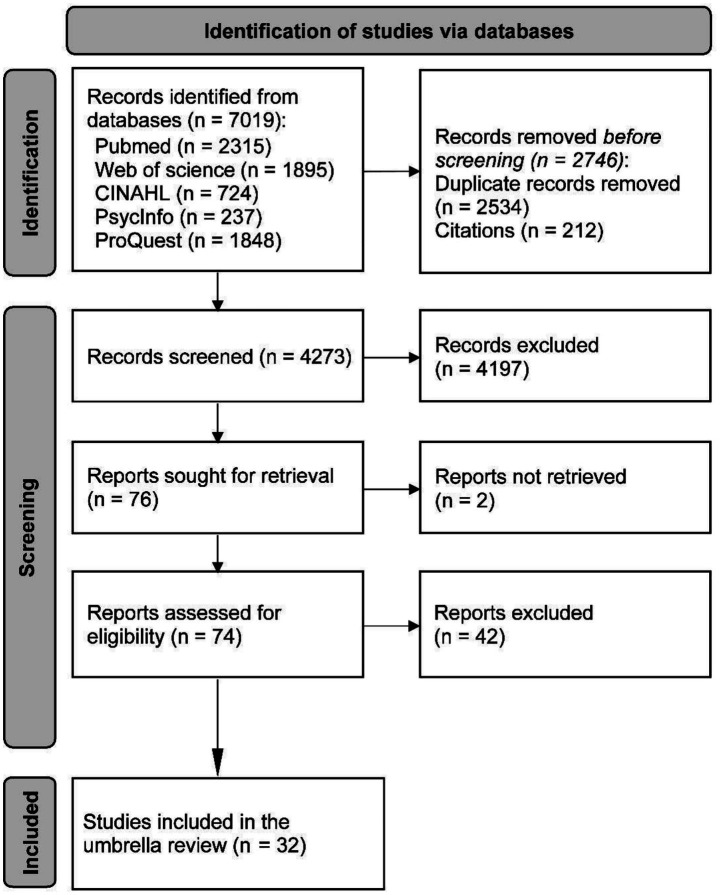
PRISMA flowchart.

A total of 7,019 articles were identified via online databases. A total of 2,534 duplicates and 212 citations were removed. After screening 4,273 articles based on title and abstract, 4,197 were excluded. Full texts were sought via institutional subscriptions and interlibrary loan; corresponding authors were also contacted when needed. Despite these efforts, two records could not be obtained and were excluded from full-text assessment (30, 31). After careful reviews of full-text articles (*n* = 74), 42 reviews were excluded. The list of excluded studies is presented in Supplementary Table S2. A total of 32 systematic reviews were included in this OoSRs (Figure 1). Reviewer agreement was high during title/abstract screening (percent agreement = 98%), full-text screening (100%), and the Google Scholar update search (100%). Given consensus resolution procedures, Cohen’s *κ* was not calculated.

### 3.2 Studies characteristics

The characteristics of the 32 studies included in this OoSRs are presented in Table 1. Systematic reviews were published between 2008 and 2024, of which 19 (60%) performed meta-analyses. Only six reviews reported an overall total sample size aggregated across included trials. Across all reviews, the median number of primary studies per review was 22 (IQR 13–32; range 4–118).

Adult-focused reviews enrolled non-athlete adults with overweight or obesity, including subgroups with metabolic syndrome or severe obesity and worksite cohorts (32–35). Reviews of children and adolescents covered preschoolers through teenagers in school, community, and outpatient/primary-care settings, often with explicit parent or family engagement (36–40). One review focused on older adults, including geriatric-obesity phenotypes, delivered in community (41). Special populations included infertile patients undergoing lifestyle programs (42) and children and adults enrolled in culturally tailored programs (43).

Programs delivered combined PA and diet intervention. PA components were typically aerobic, resistance, mixed, or High-Intensity Interval Training (HIIT), usually moderate-to-vigorous intensity, 2–5 days·week^−1^. Dietary components included hypocaloric balanced menus; Mediterranean/Dietary Approaches to Stop Hypertension (DASH); low-fat/low-carbohydrate; or meal-replacement/very-low-energy phases. Interventions were delivered by dietitians, exercise professionals, or multidisciplinary teams. Delivery ranged from fully face-to-face to hybrid or fully remote formats using web/apps, tele-monitoring, and step-count goals; several reviews contrasted supervised center-based sessions with home-based programming. Family-based delivery was common in pediatric combined PA and diet intervention and was often associated with higher adherence and more favorable anthropometric responses. Program length ranged from 2 weeks to 61 months, with most trials clustered in the 3–12-month window; higher contact frequency and ≥12-month duration was commonly associated with larger or more durable effects. In older adults, combined PA and diet intervention mitigated lean-mass and bone losses during weight reduction across 6–18 months. Control conditions included usual care/minimal advice, diet-only, exercise-only, or alternative active comparators; pediatric prevention studies typically used usual-curriculum comparators at the school or class level.

Reviews commonly reported anthropometric and body composition outcomes (weight, body mass index (BMI), BMI/BMI-for-age z-score (BMI/BMI-z), waist circumference, body-fat%), cardiometabolic markers (blood pressure, lipids, glycaemia/insulin/Homeostatic Model Assessment of Insulin Resistance (HOMA-IR)), inflammation/adipokines (C-reactive protein (CRP), Interleukin-6 (IL-6), Tumor Necrosis Factor-alpha (TNF-*α*); adiponectin/leptin), physical fitness and strength, behavioral outcomes (Moderate to Vigorous PA (MVPA), diet quality, sedentary time), quality of life/psychosocial endpoints, and, where reported, autonomic function (e.g., heart rate variability (HRV)).

Most syntheses used meta-analysis for anthropometric and cardiometabolic outcomes (e.g., long-term adult weight change and workplace BMI/waist effects), while network meta-analysis was employed to contrast multi-component nodes (e.g., face-to-face combined PA and diet intervention vs. single-component arms) in pediatric cohorts.

Across population subgroups, intervention domains showed distinct patterns. Among adults, combined PA and diet intervention reduced body weight and central adiposity and improved cardiometabolic risk, with remote/digital delivery demonstrating short-term weight loss comparable to in-person formats (12, 32, 42). In children and adolescents, family-engaged, school-centered, multi-component combined PA and diet intervention produced the most consistent improvements in BMI/BMI-z, whereas single-component PA alone rarely shifted BMI (36–38). Among older adults, resistance-inclusive combined PA and diet intervention preserved lean mass and improved strength more than diet-only or aerobic-only comparators over 8–16 weeks (41).

### 3.3 Narrative synthesis of results

Across populations and settings, SRs generally suggested that combined PA and diet intervention consistently tended to outperform single-component comparators for obesity management and prevention (11). The strength and consistency of evidence varied by outcome domain, population, and review quality, with effects appearing more consistently in longer-term interventions that featured structured or supervised delivery, higher contact frequency, and embedded behavior-change techniques such as goal setting, self-monitoring, feedback, and motivational interviewing (11, 12, 34, 35, 44).

In adults with overweight or obesity, combined PA and diet intervention was generally associated with greater and more durable reductions in body weight, BMI, and waist circumference than diet-only or PA-only strategies, with effects commonly larger in longer, higher-contact, and more structured or supervised programs (11, 35, 44). Relative to diet-only approaches, combined PA and diet intervention enhances fat-mass loss while helping preserve lean mass, which improves the quality of weight loss and simultaneously raises cardiorespiratory fitness, muscular strength, and physical function, even when incremental weight loss over diet alone is moderate (11, 32, 45, 46).

Compared to diet alone, combined PA and diet intervention showed better glycemic control, healthier lipid profiles, lower blood pressure, and favorable inflammatory and adipokine changes, including small but significant improvements in fasting glucose and insulin resistance even when additional weight loss is minimal (15, 47–49). Among adults with metabolic syndrome, combined PA and diet intervention was commonly associated with reductions in waist circumference (often ~5–15 cm) and weight loss (~3–12 kg), although estimates varied across reviews and trial designs; outcomes appeared to depend more on overall energy deficit and exercise structure than on macronutrient ratios (35).

In class II to III obesity, combined PA and diet intervention was associated with moderate but clinically relevant improvements in weight, BMI, blood pressure, triglycerides, and total or low-density lipoprotein (LDL) cholesterol, with larger effects more often reported in longer and higher-contact programs (33, 44). Over longitudinal studies (up to 15 years), multi-component lifestyle interventions approximately halve incident type 2 diabetes risk versus usual care, with greater weight loss and higher baseline fasting insulin predicting larger risk reductions (50). In adults with established type 2 diabetes, combined PA and diet intervention was associated with improvements in Glycated hemoglobin (HbA1c), fasting glucose, insulin resistance, and lipid outcomes, reinforcing therapeutic relevance beyond weight control (15).

In children and adolescents, face-to-face, family-engaged, multi-component combined PA and diet intervention appears to outperform usual care and either component alone, improving BMI, BMI-z, waist circumference, and body fat percentage at about 6 months, with higher effects when programs run at least 6–12 months and actively engage parents or the school environment (36, 37, 40, 51–54). Pre-school prevention programs rarely affect BMI, whereas management programs with active parent involvement in clinical or community settings more consistently reduce BMI or BMI-z (36, 37). In older adults, combining PA and diet intervention generates the most favorable balance of outcomes, reducing fat mass while attenuating losses of lean mass and bone typically observed with diet alone, alongside improvements in function and quality of life, while exercise-only arms improve function without meaningful weight loss (41). Workplace programs yield small to moderate improvements in BMI, waist circumference, lipids, and blood pressure, with more improvements when coaching and exercise opportunities are included rather than information-only approaches (34).

Digitally supported and telehealth modalities are associated with short-term weight loss across 8–26 weeks, particularly when delivered entirely online, although durable effects beyond the initial period depend on behavioral support, high-frequency contact, and planned follow-up (12). Evidence syntheses of culturally tailored programs indicate small weight losses in South-Asian adults and no clear anthropometric effects for school-aged children, again pointing to intensity, duration, and family engagement as effect modifiers in real-world implementation (43).

Interpretation of these patterns should be cautious given the substantial variability in methodological quality across included reviews, and the predominance of low or critically low ratings.

### 3.4 Meta-analytical results

All quantitative estimates reported below are pooled effects extracted directly from the included MAs, without re-analysis of primary trial data.

#### 3.4.1 Body weight, BMI, and weight-loss magnitude

In adults, MAs generally indicate that behavioral programs lasting at least 1 year that deliver combined PA and diet intervention tend to achieve more favorable weight outcomes than either component alone. Across 22 trials, combined PA and diet intervention decreased body weight by 1.38 kg beyond diet alone and 4.13 kg beyond exercise alone (32). Head-to-head contrasts reinforce this added value: meta-analysis of eight adult RCTs showed that, compared with PA alone, combined PA and diet intervention achieved 5.33 kg greater loss at 3 to 6 months and 6.29 kg greater loss at 12–18 months, while versus diet alone the advantage was 0.62 kg at 3 to 6 months and 1.72 kg at 12 to 18 months (11). Shorter adult programs also show benefits: across 14 trials of 8 to 26 weeks, combined PA and diet intervention reduced body weight by 2.59 kg versus usual care, with larger effects in fully online delivery and male-only trials (12). In professionally guided adult programs, 1-year absolute weight loss was only marginally smaller in adults with overweight than in those with class I or II obesity (≈1.1 kg less than class I; ≈1.5 kg less than class II), while percentage weight loss did not differ by BMI class, supporting applicability across BMI 25 to 40 kg·m^−2^ (55). In adults with class II to III obesity, pooled data across 56 studies indicated mean weight loss of ≈8.9 kg and a BMI reduction of ≈2.8 kg·m^−2^, with larger weight loss when programs lasted longer than 12 months (44), and independent reviews of severe obesity reported 1.0 to 11.5 kg more loss than controls over 3 to 24 months and BMI reductions of 0.3 to 4.0 kg·m^−2^ more than controls (33). In workplace trials in adults, pooled estimates across 13 RCTs showed BMI decreases of 0.86 kg·m^−2^ (34). In women of reproductive age with overweight or obesity, combined PA and diet intervention was associated with a mean BMI reduction of 1.42 kg·m^−2^ versus minimal care (56), and in obesity-related infertility pooled weight loss was 3.98 kg (with the best individual trials around 10 kg) (42). In South-Asian adults, culturally tailored combined PA and diet intervention reported pooled weight loss of −1.82 kg versus control, while pooled effects for BMI were not statistically significant (43). In pediatric and adolescent populations, a 6-month network meta-analysis of 118 RCTs found that face-to-face combined PA and diet intervention reduced BMI by 0.98 kg·m^−2^ and BMI-z by 0.10 SD versus control (52), while broader syntheses report a standardized mean difference of −1.09 for BMI or BMI-z at ~6 months with attenuation after contact ends (57). Pooled prevention-setting analyses reported BMI-z mean difference of −0.12 and body weight change of −1.59 kg (57), and earlier work suggested BMI reductions were approximately doubled when parents were actively engaged (36). In South-Asian school-age children, network meta-analysis did not identify a clear between-group effect for BMI or BMI-z (43).

#### 3.4.2 Waist circumference and central adiposity

In adults, combined PA and diet intervention reduced waist circumference by a further 1.68 cm versus diet and 3.00 cm versus exercise (32). In adults with class II to III obesity, waist circumference decreased but fasting glucose and HDL/LDL responses were inconsistent (44). In workplace adult trials, waist circumference decreased by 2.06 cm (34). In pediatric/adolescent RCTs, face-to-face combined PA and diet intervention reduced waist circumference by 1.49 cm versus control and outperformed face-to-face PA or diet alone for BMI and waist outcomes (37, 52). In pooled school/community prevention settings, waist outcomes improved modestly overall, though heterogeneity was high (57). In South-Asian adults, culturally tailored combined PA and diet intervention did not show significant pooled effects on waist circumference, and in South-Asian school-age children pooled waist effects were also non-significant (43).

#### 3.4.3 Fat mass, lean mass, and “quality” of weight loss

In adults, combined PA and diet intervention removed an additional 1.65 kg of fat versus diet and 3.60 kg versus exercise (32). Across adult syntheses, when structured aerobic or resistance training is delivered within combined PA and diet intervention, additional fat mass loss is ~17 to 18% from baseline, and lean-mass loss is attenuated (~2.2 to 2.5% vs. ~ 3.6% with diet alone) (46). In older adults (community-dwelling ≥60 years with obesity), diet-only groups reduced fat mass but also fat-free mass and showed small yet significant bone-density loss; combined PA and diet intervention better preserved lean tissue and attenuated bone loss (41).

#### 3.4.4 Blood pressure

In adults, diastolic blood pressure decreased by ~1.2 mm Hg versus diet and ~2.1 mm Hg versus exercise, while systolic blood pressure differences were minimal versus diet and about −2.8 mm Hg versus exercise (32). In adults with obesity and type 2 diabetes, combined PA and diet intervention was associated with modest decreases in systolic and diastolic blood pressure (15). In class II to III obesity, diastolic blood pressure fell by ~4 to 6 mm Hg and systolic pressure also declined (44), and severe-obesity reviews similarly noted modest blood pressure improvements (33). In workplace adult trials, systolic blood pressure decreased by 3.39 mm Hg and diastolic blood pressure by 2.89 mm Hg (34). In pooled school/community prevention settings, blood pressure improved in roughly one third of studies, particularly in programs ≥12 months with structured exercise plus nutrition plans (57).

#### 3.4.5 Cardiorespiratory fitness, strength, and physical function

In adults, cardiorespiratory fitness increased by ~3.6 mL·kg^−1^·min^−1^ over diet and ~2.1 mL·kg^−1^·min^−1^ over exercise (32), and broader adult syntheses report VO₂peak rises of ~2 to 5 mL·kg^−1^·min^−1^ with combined PA and diet intervention, with routine strength gains when resistance or combined training is included (46). In older adults (≥60 years with obesity), exercise-only interventions improved performance without corresponding weight loss (e.g., VO₂peak + ~ 1.4 mL·kg^−1^·min^−1^ and upper-body strength from 174 to 190 lb. one-rep-max), whereas combined PA and diet intervention produced larger VO₂peak gains (~ + 3.1 mL·kg^−1^·min^−1^) and consistent improvements in chair-rise and 6-min walk tests (41). In comparative adult work, portion-controlled diets plus ≥175 min/week combined endurance + strength training were associated with ~5% extra body-weight loss over 6 to 24 months versus usual care and outperformed diet alone by 2 to 4 kg at 6 to 12 months in programs using supervised aerobic sessions (45).

#### 3.4.6 Lipids

In adults, lipid responses favored combined PA and diet intervention over exercise alone (≈10–11 mg·dL^−1^ lower total and LDL cholesterol), while differences versus diet alone were small; HDL increased slightly versus diet (≈1.6 mg·dL^−1^) and triglycerides were modestly lower versus diet (≈10 mg·dL^−1^) (32). In the same meta-analysis of adults without diabetes, triacylglycerides decreased by −0.258 ± 0.037 mmol·L^−1^, whereas HDL showed no significant pooled change (58). In adults with obesity and type 2 diabetes, combined PA and diet intervention improved lipid profile by reducing total cholesterol and triglycerides, while HDL-C and LDL-C showed no clear pooled effect (15). In class II to III obesity, total cholesterol, LDL-C, and triglycerides decreased, with inconsistent HDL-C responses (44). In workplace adult trials, total cholesterol decreased by 6.83 mg·dL^−1^, HDL-C increased by 0.83 mg·dL^−1^, LDL-C decreased by 6.20 mg·dL^−1^, and triglycerides fell by 12 mg·dL^−1^ (34). In pediatric/adolescent RCTs, pooled estimates indicated total cholesterol SMD − 0.45 and triglycerides SMD − 0.42, with non-significant pooled effects on HDL-C and LDL-C (15), and in pooled school/community prevention settings lipids improved in roughly one third of studies (57).

#### 3.4.7 Glycemic control and insulin sensitivity

In adults, adding structured exercise within combined PA and diet intervention was associated with reductions in fasting plasma glucose of ~1.46 mg·dL^−1^ and improvements in insulin sensitivity of ~0.33 SD, despite negligible additional weight loss relative to diet alone (48). In adults without diabetes, a meta-analysis of controlled trials similarly showed that diet-plus-exercise education (≈1 year) reduced fasting glucose by −0.18 ± 0.04 mmol·L^−1^ and fasting insulin by −2.56 ± 0.58 mU·L^−1^ versus controls (58). In adults with obesity and type 2 diabetes, combined PA and diet intervention was associated with reductions in HbA1c, fasting glucose, and fasting insulin (15). In class II to III obesity, fasting glucose responses were inconsistent (44). In workplace adult trials, fasting glucose decreased by 1.23 mg·dL^−1^ (34). In pediatric/adolescent RCTs, pooled effects indicated improved glycemic control (HbA1c SMD − 0.52; fasting insulin SMD − 0.32), with a large but imprecise reduction in fasting glucose (15), and pooled school/community prevention settings also reported improvements in a subset of studies (57).

#### 3.4.8 Inflammation and adipokines

In adults, pooled meta-analytic estimates suggested small inflammatory effects when combined PA and diet intervention was contrasted with diet-only approaches (CRP SMD − 0.16, 95% CI − 0.29 to −0.03), while IL-6 and TNF-*α* pooled effects were near null (49). In adults with obesity and type 2 diabetes, pooled analyses suggested rises in adiponectin of ~1.4 μg·mL^−1^ and reductions in leptin of ~7.3 ng·mL^−1^ (15), while broader syntheses similarly report small CRP reductions with pooled null effects on IL-6 and TNF-α (15, 47, 49). In pediatric/adolescent RCTs, inflammation markers showed generally favorable pooled changes in limited trials (e.g., CRP SMD − 0.46) with no clear pooled change in TNF-α (15), and longitudinal cohorts reported endocrine adaptations tracking weight trajectories (e.g., leptin falling with loss and rising with regain; TSH and FT3 predicting later regain) (53).

#### 3.4.9 Physical activity and dietary behavior change (process outcomes)

In adults, PA indices were not consistently higher at 12 months in combined PA and diet intervention arms, yet weight and fitness outcomes still favored the dual-component approach (11). In pooled school/community prevention settings, PA increased by at least 10% in about half of studies assessing it, and dietary intake improved by ~41%, with stronger effects in programs of at least 12 months featuring structured exercise plus nutrition plans (57). In school-centered pediatric programs, classroom and after-school initiatives increased moderate-to-vigorous activity (e.g., ~26 extra minutes/day in APPLE; ~47 min of active time in after-school free play) and improved dietary choices (e.g., reduced fizzy-drink intake; higher fruit selection), although BMI changes were not universal (54).

#### 3.4.10 Quality of life, functional outcomes, and adverse events

In older adults (≥60 years with obesity), quality of life (when reported) improved more in combined PA and diet intervention arms, with SF-36 physical composite rising by ~8 to 15% compared with ~3% in diet-only and no change in control; adverse events were generally minor (typically transient musculoskeletal pain) (41). In pediatric contexts, family-based outpatient training improved health-related quality of life at 12 months (53).

#### 3.4.11 Long-term disease incidence

Over a median 13-year follow-up in adults, combined PA and diet intervention was associated with an approximately 50% lower incidence of type 2 diabetes versus usual care (odds ratio ~0.44), with larger risk reductions accompanying greater weight loss and higher baseline fasting insulin (50).

### 3.5 The methodological quality assessment

The AMSTAR 2 appraisal of 32 systematic reviews showed substantial variation in methodological rigor, with important implications for confidence in the synthesized findings (Supplementary Table S3). Using the decision rules, overall confidence ratings were High 10/32 (31.2%), Moderate 2/32 (6.2%), Low 10/32 (31.2%), and critically low 10/32 (31.2%), with approximately 69% of reviews rated as low to critically low (22/32, 68.8%). For the critical domains, preregistration or *a priori* protocol (Item 2): Y 10/32 (31.2%); N 22/32 (68.8%); PY 0/32 (0.0%). Comprehensive multi-database searching (Item 4): Y 23/32 (71.9%); N 1/32 (3.1%); PY 8/32 (25.0%). Transparency around study exclusion remained a consistent weakness, few reviews listed excluding studies with reasons (Item 7: Y 8/32, 25.0%; N 24/32, 75.0%; PY 0/32, 0.0%). Study-level risk of bias was assessed in over half of reviews (Item 9: Y 18/32, 56.2%; N 13/32, 40.6%; PY 1/32, 3.1%). Among reviews that conducted a meta-analysis (denominator = Y + N + PY = 23), statistical methods were almost always appropriate (Item 11: Y 22/23, 95.7%; N 1/23, 4.3%; PY 0/23, 0.0%), yet incorporation of risk of bias into interpretation was inconsistent (Item 13: Y 11/32, 34.4%; N 13/32, 40.6%; PY 8/32, 25.0%). Publication-bias diagnostics were common but not universal in meta-analyses (Item 15: Y 17/23, 73.9%; N 5/23, 21.7%; PY 1/23, 4.3%). Overall, critical weaknesses most frequently related to the absence of preregistered protocols, limited transparency around excluded studies, and inconsistent integration of risk of bias into result interpretation.

Regarding non-critical domains, most reviews framed a clear PICOS question (Item 1: Y 31/32, 96.9%; N 0/32, 0.0%; PY 1/32, 3.1%) and justified eligible study designs (Item 3: Y 29/32, 90.6%; N 1/32, 3.1%; PY 2/32, 6.2%). Duplicate processes were less consistently reported, particularly for study selection (Item 5: Y 23/32, 71.9%; N 5/32, 15.6%; PY 4/32, 12.5%) and data extraction (Item 6: Y 21/32, 65.6%; N 6/32, 18.8%; PY 5/32, 15.6%). Descriptions of included studies were typically detailed (Item 8: Y 31/32, 96.9%; N 0/32, 0.0%; PY 1/32, 3.1%), while reporting of primary-study funding was rare (Item 10: Y 1/32, 3.1%; N 31/32, 96.9%; PY 0/32, 0.0%). The extent to which the impact of risk of bias on results was examined varied (Item 12: Y 11/32, 34.4%; N 12/32, 37.5%; PY 9/32, 28.1%). Statistical heterogeneity was addressed where applicable (Item 14: Y 22/32, 68.8%; N 2/32, 6.2%; PY 3/32, 9.4%). Finally, review-level funding and conflicts of interest were usually disclosed (Item 16: Y 29/32, 90.6%; N 2/32, 6.2%; PY 1/32, 3.1%).

### 3.6 Overlap of the included studies

Overlap results is presented in Supplementary Table S4 and illustrated heatmap for each outcome in Supplementary Figures S1–S10 Across outcome domains, CCA values ranged from 0.0 to 0.6%, indicating slight overlap. The BMI or body weight domain (28 reviews, 715 total study instances, 647 unique studies) showed a CCA of 0.4%, reflecting minimal duplication of primary trials. Similarly small overlaps were observed for waist circumference (0.2%), body composition (0.1%), metabolic markers (0.6%), and blood pressure (0.3%). Physical fitness and function showed a CCA of 0.2%, still within the slight category. Several domains exhibited zero overlap, including quality of life, dietary behavior, and inflammatory markers. However, in domains with few reviews or limited primary study pools, a CCA of 0.0% likely reflects limited opportunity for overlap rather than comprehensive coverage of the evidence base. Overall, the overview is therefore at low risk of double counting across reviews, supporting cautious synthesis of findings across reviews.

Given the generally low methodological quality of many included SRs, these findings should be interpreted conservatively, emphasizing patterns that are consistent across higher-quality reviews and outcomes that are supported by multiple independent syntheses.

### 3.7 Summary of evidence

A synthesis of 32 systematic reviews shows that multicomponent programs combining structured PA with an energy-restricted or quality-improving diet tend to achieve more favorable outcomes than single-component approaches across the life course.

In adults, reviews consistently report larger reductions in weight, BMI, waist circumference, and fat mass in interventions lasting at least 12 months compared with diet-only or exercise-only strategies, with exercise contributing to improved weight-loss quality through better preservation of lean mass and fitness (32, 46). Cardiometabolic risk markers generally improve modestly yet consistently, including fasting glucose, insulin resistance, CRP, and adipokines, and long-term programs are associated with a substantially lower incidence of type 2 diabetes (35, 47, 48, 50).

In youth, face-to-face, family-engaged diet-plus-activity interventions are most often associated with more durable improvements BMI and BMI-z changes, whereas brief or single-component formats commonly show attenuation of effects in the absence of maintenance (15, 37, 49, 52).

In older adults, hypocaloric diets combined with supervised, resistance-inclusive exercise are associated with favorable trade-offs between weight reduction, preservation of lean mass, limiting sarcopenia, and improvements in function and quality of life (41). Evidence from specific settings, including infertility care, workplaces, and digitally supported or hybrid programs, suggests modest but clinically meaningful short-term benefits, typically on the order of 2–3 kg of weight loss (12, 34, 42, 56).

Across populations, more favorable outcomes are reported more often in combined diet and PA programs of longer duration (≥6–12 months), characterized by higher contact frequency, supervised or structured delivery, inclusion of resistance training, family engagement in youth, and maintenance of an overall energy deficit (40).

These findings remain clinically useful, but they should be interpreted as reflecting an evidence base dominated by traditional lifestyle-intervention models; contemporary obesity science increasingly suggests that treatment response may also be shaped by gut-derived biology, including satiety signaling, incretin pathways, and microbiome-related differences in host energy handling (59, 60).

## 4 Discussion

This overview indicates that combined PA and diet intervention tends to outperform single-component approaches across weight, adiposity, body-composition quality, fitness, and cardiometabolic risk markers. In adults, combined PA and diet intervention lasting at least 12 months produces larger reductions in weight, BMI, waist, and fat mass than diet-only or exercise-only, and adding exercise preserves lean mass and improves fitness, especially with resistance training. Cardiometabolic markers improve modestly but consistently, driven more by sustained energy deficit and structured exercise than by macronutrient composition, and longer combined PA and diet intervention is associated with lower type 2 diabetes incidence. In youth, face-to-face, family-engaged combined PA and diet intervention yields the largest and most durable BMI gains, while brief or single-component efforts often fade. Benefits are also seen in older adults and in workplace and digital or hybrid settings, typically 2 to 3 kg short-term, and are strongest when contact is high, sessions are structured or supervised, resistance training is integrated, families are engaged, and an energy deficit is maintained.

These findings should also be interpreted relative to the current hierarchy of obesity treatments. Although combined diet and physical activity interventions generally outperform single-component lifestyle approaches, the absolute magnitude of average weight loss in this overview is typically modest compared with modern incretin-based pharmacotherapy and metabolic bariatric surgery. In contemporary randomized trials, semaglutide 2.4 mg and tirzepatide have produced substantially greater mean weight reductions than those typically seen in the lifestyle reviews included here, while current bariatric-surgery guidance continues to regard metabolic bariatric surgery as the most effective durable treatment for severe obesity (16, 17). Accordingly, pharmacological and surgical approaches generally achieve larger average weight-loss magnitudes, while combined PA and diet interventions remain essential as foundational or complementary strategies that support cardiometabolic health, functional capacity, and long-term weight management. Dietary research also increasingly suggests that food processing and dietary composition may influence appetite regulation and weight change through gut-derived pathways, including endogenous GLP-1 signaling, although this evidence remains evolving rather than definitive (6, 7).

Taken together, these patterns suggest that, within the lifestyle reviews included in this overview, intervention features such as duration, contact frequency, supervision, resistance training, and maintenance support were more consistently associated with favorable outcomes than differences in reported macronutrient composition alone (32, 35, 46). This does not imply that dietary composition or gut-derived biology is unimportant; rather, the available review-level evidence was better able to distinguish the contribution of program structure than the contribution of specific mechanistic pathways, including satiety signaling, incretin responses, or microbiome-related differences in host energy handling (6, 59–62). This apparent discrepancy reflects differences in explanatory level. Indeed, while energy imbalance remains a necessary condition for weight change, intervention-level outcomes appear to be more strongly influenced by behavioral and structural factors that determine adherence, implementation, and long-term sustainability.

Accordingly, the present overview supports the importance of sustained, well-designed multicomponent lifestyle programs. At the same time, these findings should be interpreted within a broader and evolving biological framework, in which treatment response is understood to arise from interactions between behavior, diet quality, and underlying physiological processes. In this context, combined lifestyle interventions may operate primarily through behavioral and energy-balance pathways, but their effectiveness may also be modulated by individual biological responsiveness, including gut–brain signaling and microbiome-related mechanisms, although these pathways were not directly assessed in the included reviews and are supported primarily by emerging experimental and translational evidence (6, 59–62).

Where mechanistic explanations are discussed below, these should be interpreted as plausible interpretations consistent with observed patterns across the included systematic reviews, rather than as direct causal inferences from primary experimental evidence.

Relative to diet alone, combined PA and diet intervention produced small to moderate additional reductions in body weight and waist circumference, with larger advantages over PA alone, and parallel improvements in blood pressure and cardiorespiratory fitness. These patterns are consistent with head-to-head meta-analytical contrasts reported in the Results section, indicating modest but clinically relevant additional weight loss relative to single-component interventions, together with small reductions in diastolic blood pressure and meaningful gains in VO₂max (11, 32). Although the kilogram differences are modest, the clinical gain is larger because exercise appears to improve the quality of weight loss, preserves lean mass, and stabilizes resting energy expenditure, which supports function and may reduce weight-regain risk; the stronger advantage over activity-only arms underscores that diet supplies the primary energy deficit while exercise shapes composition and fitness (11, 32, 46). This pattern is consistent with the interpretation that the added benefit of combined PA and diet intervention may reflect the contribution of supervised, structured exercise alongside a sustained dietary energy deficit, rather than changes in self-reported PA alone.

These findings should also be interpreted in the context of the rapidly evolving obesity-treatment landscape. Most reviews included in this overview synthesized trials developed before the current era of highly effective incretin-based anti-obesity pharmacotherapies. In contemporary trials, semaglutide 2.4 mg produced a mean body-weight reduction of 14.9% at 68 weeks, while tirzepatide produced mean reductions approaching 20% at 72 weeks, depending on dose (16, 17). Accordingly, our overview should not be interpreted as suggesting that combined diet-plus-physical-activity interventions achieve weight-loss magnitudes comparable to newer pharmacological therapies. Rather, the present evidence supports lifestyle intervention as a foundational treatment strategy that improves body composition, fitness, and cardiometabolic risk, and that remains relevant whether used alone or alongside pharmacotherapy, which is itself indicated as an adjunct to a reduced-calorie diet and increased physical activity (16, 17).

Importantly, short-term digital and hybrid combined PA and diet intervention also revealed clinically meaningful weight loss over 8 to 26 weeks, and the superiority of combined PA and diet intervention was similar across BMI strata, supporting generalizability from BMI 25 to 40 kg·m^−2^ in professionally guided settings (12, 55). Digital delivery appears effective when contact is frequent and structured, but without planned maintenance weight commonly drifts back as coaching intensity falls, mirroring attenuation after face-to-face combined PA and diet intervention once support ends (11, 12).

In severe obesity, combined PA and diet intervention produced clinically significant weight loss, approximately 9 kg on average, and improved cardiometabolic profiles, with effect sizes increasing when contact was more frequent and program duration exceeded 12 months (33, 44). For people with higher BMI, longer combined PA and diet intervention with more frequent support helps the body adapt and improve movement. Supervised sessions that include strength training build function and can also make future treatments more effective if needed (33, 44, 46).

Otherwise, workplace implementations demonstrated moderate but consistent benefits on BMI, waist circumference, and lipids, underscoring feasibility of combined PA and diet intervention in real-world delivery models that include coaching and structured activity rather than information alone (34). These effects are modest per person but meaningful at population scale, suggesting that even relatively small individual-level benefits may translate into relevant public-health gains when implemented broadly, provided that sustained engagement, maintenance support, and enabling system-level conditions are in place. Such effects also depend on behavioral scaffolding such as coaching, self-monitoring, and structured activity, since information-only approaches rarely move BMI or lipids (34).

Over longer horizons, combined PA and diet intervention halved incident type 2 diabetes risk compared with usual care, indicating durable disease-prevention potential even as between-group weight differences attenuate with time (50). This durability points to weight-independent pathways, improved insulin sensitivity, and higher cardiorespiratory fitness as mediators beyond early weight loss (15, 50).

Within combined PA and diet intervention, structured exercise alongside a hypocaloric diet improved fasting glucose by roughly 1.46 mg·dL^−1^ and insulin resistance by about one third of a standard deviation despite negligible extra weight loss, and it shifted adipokines toward a more cardioprotective profile (lower leptin and higher adiponectin) (48). Pooled inflammation signals showed a small reduction in C-reactive protein, with near-null average effects for IL-6 and TNF-*α*. In adults with obesity and type 2 diabetes, combined PA and diet intervention reduced HbA1c, fasting glucose, fasting insulin, triglycerides, and blood pressure, with standardized effects in the small to moderate range across outcomes (15, 47). Clinically, these findings support consideration of progressive, structured exercise as part of combined PA and diet intervention, even when extra weight loss is expected to be small, since glycemic control and blood pressure can still improve through exercise-mediated insulin action and vascular effects (15, 48).

Program outcomes tended to improve more consistently with longer duration and higher contact frequency, as combined PA and diet intervention lasting 6–12 months with regular structured sessions produced larger and more lasting effects than shorter or low-contact formats (35, 40, 55). Supervision and structure, particularly the deliberate inclusion of resistance training, were repeatedly linked to superior body-composition and functional outcomes, including better preservation of fat-free mass and larger gains in strength and cardiorespiratory fitness compared with diet alone or aerobic-only exercise (41, 46). In applied settings, workplace and digital combined PA and diet intervention worked best when coaching, self-monitoring, and feedback were built in; fully online delivery achieved short-term losses comparable to in-person models when contact was frequent, but maintenance strategies were essential to limit post-program regain (12, 34). A practical sequence is to first establish an appropriate energy-deficit diet, then add structured exercise including resistance training two to three times per week, and finally build behavior-change support through goal setting, self-monitoring, feedback, and a planned maintenance phase after 3–6 months (11, 35, 46).

Importantly, however, confidence in these conclusions varies across outcome domains. Where higher-quality systematic reviews (AMSTAR 2 high or moderate confidence) were available, particularly for adult weight, adiposity, and fitness outcomes, conclusions are comparatively more robust. In contrast, findings related to some metabolic, inflammatory, and culturally tailored combined PA and diet intervention are derived predominantly from low or critically low-quality reviews and should therefore be interpreted cautiously as provisional rather than definitive.

From an implementation perspective, the evidence synthesized in this overview suggests that effective combined PA and diet intervention is most commonly characterized by dietary energy restriction or quality-improving dietary counseling alongside structured PA; a duration of at least 6–12 months; regular professional contact with supervised or structured sessions; inclusion of resistance-based exercise alongside aerobic activity; and a planned maintenance phase incorporating self-monitoring and booster contacts.

The pediatric pattern echoed adult results but was more sensitive to delivery details. Face-to-face, family-engaged combined PA and diet intervention outperformed usual care and single-component comparators for BMI, BMI-z, waist circumference, and body fat percentage at approximately 6 months, whereas school-based prevention alone often produced small and heterogeneous anthropometric effects. Sustained benefits typically required at least 6 to 12 months of contact with explicit parent involvement, and weight regain was common once support ceased, highlighting the need for planned maintenance and environmental scaffolding in homes and schools (36, 37, 52, 53, 57). Family engagement likely enhances adherence through home meal structure, healthier food availability, and logistics for organized activity, while school-only prevention tends to underdose intensity and contact, which blunts BMI effects; programs should therefore combine parent components with school or community supports for at least one academic year (36, 37, 52).

Culturally tailored combined PA and diet intervention for South-Asian adults yielded small average weight losses, suggesting that tailoring improves acceptability but does not substitute for adequate intensity and duration; in South-Asian school-age children, controlled evidence did not show clear anthropometric benefits, again pointing to dose and family engagement as key effect modifiers (43). Tailoring should be layered on top of sufficient duration and contact rather than seen as a replacement for program dose (43).

Two additional clinical subgroups warrant emphasis. In older adults with obesity, combined PA and diet intervention was consistently superior to diet alone or aerobic-only training for preserving lean mass and bone while improving strength, function, and quality of life; exercise alone improved function but did not reduce weight, highlighting the complementary roles of both components in this population (41). For older adults, resistance training is pivotal to protect muscle and bone during energy restriction; reported adverse events were minor, supporting scalability in community settings (41).

Among individuals with obesity-related infertility and women of reproductive age, combined PA and diet intervention improved body weight and BMI, with some trials reporting larger individual losses and ancillary reproductive benefits in the broader narrative, which strengthens the case for integrating combined PA and diet intervention into fertility care pathways (42, 56). Given low risk and broader cardiometabolic gains, combined PA and diet intervention can run in parallel with fertility care and may improve responsiveness through weight and insulin-sensitivity pathways (42).

The apparent superiority of combined diet and physical activity interventions should not be reduced to a simplistic “calories in versus calories out” model. While sustained dietary energy deficit and structured exercise remain central to the effects summarized in this overview, emerging evidence suggests that obesity-related responses are also shaped by gut-derived mechanisms, including enteroendocrine signaling, endogenous GLP-1 responses, bile-acid pathways, and gut-microbiome-related influences on satiety and metabolizable energy (6, 7, 62, 63). However, these mechanisms should be interpreted as plausible complementary explanations rather than definitive drivers of the observed effects. Controlled human data indicate that diet–microbiome interactions can alter host metabolizable energy, and recent reviews further suggest that modern dietary and pharmacological obesity treatments interact with gut–brain and gut–microbiome pathways (6, 7, 62, 63). Similarly, the metabolic benefits of Roux-en-Y gastric bypass are increasingly understood to involve more than restriction or malabsorption alone; rapid nutrient delivery to the distal intestine is associated with marked postprandial increases in GLP-1 and related gut peptides, alongside other neurohormonal and metabolic adaptations (62, 63). Nevertheless, these pathways were not directly assessed in the primary studies included in this overview, and their contribution to the observed effects cannot be determined. Accordingly, interpretation is limited to comparative effectiveness patterns rather than mechanistic attribution. Future trials should also examine whether combining exercise with higher-quality dietary patterns influences immunometabolic outcomes through pathways beyond weight loss alone, including inflammation, appetite regulation, gut-derived signaling, and microbiome-related metabolites.

Implementation implications follow directly from these patterns. Across adult strata, including metabolic syndrome and higher BMI classes, outcomes tracked more closely with total energy deficit, exercise structure and supervision, and behavior-change components than with macronutrient composition per se within combined PA and diet intervention frameworks. This supports prioritizing adherence-friendly, culturally acceptable dietary patterns paired with progressive, resistance-inclusive PA rather than seeking marginal gains from macronutrient tweaking alone (27, 33, 44, 64). Health systems and employers can scale access through hybrid and online delivery, but should retain coaching, self-monitoring, and feedback loops that drive adherence and outcomes; workplace evidence already demonstrates that such scaffolding is associated with better anthropometric and lipid changes than information-only approaches (12, 34). Health systems can scale access with hybrid models if they preserve coaching and monitoring, and employers can integrate step goals, supervised sessions, and nutrition support to move beyond information-only approaches (34).

In pediatrics, services should privilege combined PA and diet intervention with family engagement, sufficient duration and intensity, and school-environment changes, and avoid reliance on PA-only strategies for BMI change (36, 37, 40, 52). For fertility care, integrating combined PA and diet intervention offers a pragmatic, low-risk adjunct that improves BMI and may enhance reproductive endpoints in broader care pathways (42).

Meta-analytic evidence clarifies both durability and maintenance needs: in adults, resistance-inclusive combined PA and diet intervention outperforms single-component approaches for 12–18 months, and combined PA and diet intervention confers a ~ 56% relative reduction in incident type 2 diabetes on long-term follow-up, whereas in children and adolescents, BMI/BMI-z improvements seen at ~6 months often attenuate within 6–12 months after contact ends (11, 32, 37, 50, 57). Accordingly, maintenance should be planned from the outset, using low-burden booster contacts, continued self-monitoring, environmental supports, and periodic supervised blocks, to sustain fitness and limit regain; especially in youth where withdrawal of support often precedes relapse (11, 37).

Building on this evidence, clinicians may consider prioritizing combining PA and diet intervention with supervised resistance training, frequent contact, and relapse-prevention plans for adults with overweight or obesity, including those with type 2 diabetes or metabolic syndrome (15, 35). For older adults, resistance training is even more critical to preserve musculoskeletal health during weight reduction (41). Health systems and employers should leverage hybrid or online delivery to scale access, while retaining coaching, self-monitoring, and feedback loops that drive adherence and outcomes (12, 34). In pediatrics, policies and services should privilege combined PA and diet intervention with family engagement, sufficient duration and intensity, and school-environment changes while avoiding reliance on PA-only approaches for BMI change (36, 37, 52). Finally, for fertility care in people with obesity, integrating combined PA and diet intervention improves BMI (42).

These cardiometabolic benefits should also be interpreted within the rapidly evolving obesity-treatment landscape. Although combined physical activity and diet interventions improve glycaemic control, lipid profiles, blood pressure, and selected inflammatory markers, the absolute magnitude of average weight loss is generally modest compared with contemporary incretin-based pharmacotherapy and metabolic bariatric surgery (16, 17, 63). Emerging dietary evidence further suggests that replacing ultra-processed foods with minimally processed dietary patterns can improve weight and cardiometabolic health, although the mechanistic contribution of endogenous GLP-1 and related gut-derived pathways remains an active area of investigation rather than a settled explanation (6, 7, 62). Accordingly, the scalability of the present findings should be understood in the context of an evidence base composed largely of traditional lifestyle trials that were not designed to compare directly with modern GLP-1 receptor agonists, bariatric procedures, or microbiome-focused dietary strategies. At the same time, this gap highlights an important research priority, namely the direct evaluation of combined exercise with minimally processed, fiber-rich, polyphenol-rich dietary patterns using integrated immunometabolic, gut-hormone, and microbiome outcomes (62, 65, 66).

### 4.1 Methodological quality considerations

The AMSTAR 2 appraisal indicates that while a substantial evidence base exists, confidence in specific effect estimates varies considerably across outcome domains. Primary conclusions of this overview are therefore driven mainly by systematic reviews rated as moderate to high quality, particularly in adult populations, and should be interpreted cautiously where evidence is derived predominantly from low or critically low-quality reviews. The AMSTAR 2 appraisal further indicates marked variability in review conduct and reporting across the 32 systematic reviews, with overall confidence skewed toward the lower end of the scale. Ratings were High 10/32 (31.2%), Moderate 2/32 (6.2%), Low 10/32 (31.2%), and critically low 10/32 (31.2%). This distribution warrants caution when interpreting pooled or narrative conclusions derived from the corpus, as multiple critical weaknesses, rather than minor reporting lapses, drove most downgrades.

Three items emerge from the critical domains. First, protocol preregistration (Item 2) was uncommon (10/32 “Yes” [31.2%]; 22/32 “No” [68.8%]), which heightens the risk of *post hoc* decision making (e.g., selective eligibility criteria and flexible outcome handling) and undermines reproducibility. Routine registration (e.g., PROSPERO or OSF), with timestamped, prespecified methods, should be considered foundational. Second, transparency of study exclusion (Item 7) was poor (8/32 “Yes” [25.0%]; 24/32 “No” [75.0%]), limiting auditability of the search-to-inclusion pipeline and raising concerns about selection bias. Publishing a PRISMA-compliant exclusions table with reasons should be standard practice. Third, while study-level risk of bias assessment (Item 9) was performed in over half of reviews (18/32 “Yes” [56.2%]; 13/32 “No” [40.6%]; 1/32 “Partial Yes” [3.1%]), its integration into interpretation (Item 13) was inconsistent (11/32 “Yes” [34.4%]; 13/32 “No” [40.6%]; 8/32 “Partial Yes” [25.0%]). This disconnect likely weakens inferential strength even when risk of bias is assessed, because credibility judgments are not propagated into the analytic or narrative synthesis (e.g., via sensitivity analyses, stratification, or explicit downgrading of confidence).

Where meta-analyses were conducted (23/32 reviews; 71.9%), statistical methods were almost always appropriate (Item 11: 22/23 “Yes” [95.7%]), and publication-bias diagnostics were attempted in most cases (Item 15: 17/23 “Yes” [73.9%]). These are notable strengths. However, small-study bias tests are underpowered when k is small, and their absence in roughly a quarter of meta-analyses, combined with weak protocol use and exclusion-list transparency, means residual reporting and selection biases cannot be ruled out. Moreover, even technically sound meta-analysis cannot compensate for upstream shortcomings in protocolization, selection transparency, or the failure to carry risk-of-bias judgments into conclusions.

The non-critical domains were stronger. Almost all reviews framed clear PICOS questions (Item 1: 31/32 “Yes” [96.9%]; 1/32 “Partial Yes” [3.1%]) and described included studies in detail (Item 8: 31/32 “Yes” [96.9%]; 1/32 “Partial Yes” [3.1%]). Most justified eligible designs (Item 3: 29/32 “Yes” [90.6%]), and heterogeneity was commonly addressed where applicable (Item 14: 22/32 “Yes” [68.8%]; 2/32 “No” [6.2%]; 3/32 “Partial Yes” [9.4%]). Nevertheless, duplicate processes were not universal: study selection was performed in duplicate in 23/32 (Item 5: 71.9%) and duplicate data extraction in 21/32 (Item 6: 65.6%), leaving room for avoidable screening and transcription errors. Most strikingly, only 1/32 reviews reported funding sources of the primary studies (Item 10: 3.1%), a pervasive gap that limits assessment of sponsor influence and constrains bias-related sensitivity analyses.

Collectively, these patterns suggest that the credibility of effect estimates in this literature is constrained less by analytic technique and more by design transparency, selection accountability, and evidence-appraisal practice. For decision makers; and for any umbrella review or overview drawing on this corpus; two safeguards are advisable: (i) weight conclusions toward reviews with moderate to high AMSTAR 2 confidence and better compliance with critical items, and (ii) conduct sensitivity/best-evidence syntheses that down-weight or explicitly flag findings dominated by critically low reviews, especially where conclusions hinge on small, heterogeneous, or industry-funded primary evidence.

### 4.2 Limitations of the evidence base and of this OoSRs

This overview has several limitations. Our findings rely on the quality of 32 source reviews, which varied substantially: approximately 31% were high quality and roughly 69% were low or critically low; 69% were not preregistered, 75% did not list excluded studies, and only 34% formally incorporated risk-of-bias; any of which may have influenced pooled or narrative conclusions. We did not apply GRADE at the overview level because methods are unsettled, so we cannot formally rate certainty. Primary studies differed substantially in intervention length, contact frequency, staffing, exercise type and dose (aerobic, resistance, HIIT), diet approach (balanced hypocaloric, Mediterranean/DASH, lower-fat/carbohydrate, meal replacements), and delivery (in-person vs. digital), making it hard to credit specific components and pushing some reviews toward narrative summaries. Outcomes were defined and measured on different schedules (e.g., BMI vs. BMI-z in youth; mixed fitness tests), which complicates harmonization and can inflate variability. Durability is a concern: several reviews reported that benefits fade 6–12 months after programs end, highlighting the need for maintenance or booster plans and longer follow-up. Although overall overlap across reviews was small (corrected covered area 0.0–0.6%), very low overlap in thinly covered topics likely reflects limited coverage rather than true independence, so some duplication or gaps may remain. Selective reporting and publication bias cannot be ruled out; bias tests were not universal, funding of primary studies was rarely reported, and adverse events and cost outcomes were sparse and inconsistent. Implementation factors (adherence, fidelity) were inconsistently measured; often by self-report; and were seldom linked to outcomes; cost reporting was limited. Generalizability is uneven: while there is evidence for severe obesity, older adults, and infertility, data are thin in low-resource settings and among adolescents in transition. Finally, we used the effect estimates as reported by each systematic review; without reanalyzing primary data, any analytic choices or errors in those reviews carry into this overview. An additional limitation is that most included reviews synthesized traditional lifestyle trials conducted before the current GLP-1RA era and outside direct comparison with metabolic bariatric surgery or minimally processed-food interventions; therefore, this overview is better suited to evaluating the relative effectiveness of combined versus single-component lifestyle strategies than to ranking lifestyle treatment against contemporary pharmacological, surgical, or gut-microbiota-oriented dietary approaches.

## 5 Conclusion

Across the included systematic reviews, combined physical activity and diet interventions generally demonstrated more favorable outcomes than single-component strategies, with the most consistent benefits observed in programs that were longer, structured, supervised, and supported by behavior-change strategies, and, in youth, included active family engagement. Although the average magnitude of weight loss was usually modest, combined interventions contributed importantly to improved body-composition quality, fitness, and cardiometabolic health, with some benefits extending beyond additional weight loss alone. Their value may therefore lie less in producing large absolute weight reductions than in improving a multicomponent behavioral and clinical foundation for long-term obesity care. At the population level, even modest individual effects may still be relevant when delivered at scale, although their public-health impact is likely to depend on sustained engagement, maintenance support, and enabling system-level conditions.

In contemporary obesity management, lifestyle intervention and pharmacotherapy should not be viewed as competing options; rather, structured physical activity and dietary support remain foundational components of obesity care, and are increasingly used alongside anti-obesity medications to enhance weight reduction, improve cardiometabolic health, and support long-term weight maintenance. At the same time, contemporary pharmacological and surgical treatments generally achieve substantially larger average weight loss than the lifestyle-only interventions synthesized here, which reinforces the importance of interpreting lifestyle treatment as a foundational and complementary component of obesity care rather than a direct substitute for higher-efficacy therapies.

Future work should prespecify and transparently report behavior-change components and contact dose to enable dose–response analyses; standardize core outcome sets (e.g., BMI and BMI-z, waist circumference, body-fat percentage, VO₂peak, HbA1c, HOMA-IR) and include objective device-based activity and sedentary metrics; use dismantling and factorial designs to isolate the incremental value of resistance training, supervision, and digital features; extend follow-up with explicit maintenance and relapse-management strategies; report cost-effectiveness to inform scaling decisions; and improve representation of older adults, adolescents, and underserved groups. Future studies should also examine whether combining exercise with minimally processed, fiber-rich, higher-quality dietary patterns yields additional immunometabolic benefits, including effects on appetite-related, gut-derived, and microbiota-related pathways, as the evidence base becomes more mature.

## Data Availability

The original contributions presented in the study are included in the article/Supplementary material, further inquiries can be directed to the corresponding author/s.

## Footnotes

1
https://github.com/thdiakon/ccaR
