# Supplementary material for: Combined diet and physical activity effects on health-related outcomes in people with overweight or obesity: an overview of systematic reviews
Source: Front Nutr. 2026 May 13;13:1821389. doi: 10.3389/fnut.2026.1821389 (PMC13212185; doi:10.3389/fnut.2026.1821389)
Supplement: Supplementary file 1 [file Table_1.DOCX]

Supplementary Material

# Supplementary Figures and Tables

## Supplementary Tables

Table S1. Search syntax for each database.

| **Database** | **Syntax** |
| --- | --- |
| **PubMed**  **+**  **Human filter**  **Exclude preprint**  **2315** | (((multi-domain[tw] OR bi-domain[tw] OR multidomain[tw] OR bidomain[tw] OR lifestyle[tw] OR multifaceted[tw] OR multidisciplinary[tw] OR crossdisciplinary[tw] OR interdisciplinary[tw] OR holistic[tw] OR multi-component[tw] OR multicomponent[tw] OR multi-dimensional[tw] OR multidimensional[tw] OR multi-factorial[tw] OR multifactorial[tw]) AND intervention*)  AND (Obesity[MeSH] OR obesity[tw] OR "Body Mass Index"[MeSH] OR "body mass index"[tw] OR BMI[tw] OR "Blood Pressure"[MeSH] OR "blood pressure"[tw] OR "Hypercholesterolemia"[MeSH] OR Hypercholesterolemia[tw] OR "high cholesterol"[tw] OR "Cardiovascular Diseases"[MeSH] OR "cardiovascular disease"[tw] OR "Metabolic Syndrome"[Mesh] OR "Metabolic Syndrome"[tw]))  AND ("Meta-Analysis as Topic"[MeSH] OR "systematic review"[tw] OR "systematic literature review"[tw] OR "meta-Analys*"[tw] OR "systematic review of the literature"[tw] OR "meta-analytical review"[tw] OR "systematic review and meta-analys*"[tw] OR "systematic review with meta-analys*"[tw]) |
| **Web of Science**  **1895** | TS=(((multi-domain OR bi-domain OR multidomain OR bidomain OR lifestyle OR multifaceted OR multidisciplinary OR crossdisciplinary OR interdisciplinary OR holistic) AND intervention*)  AND (obesity OR "body mass index" OR BMI OR "blood pressure" OR Hypercholesterolemia OR "high cholesterol" OR "cardiovascular disease" OR "Metabolic Syndrome")  AND ("Meta-Analysis as Topic" OR ("systematic review" OR "systematic literature review" OR "meta-Analys*" OR "systematic review of the literature" OR "meta-analytical review" OR "systematic review and meta-analys*" OR "systematic review with meta-analys*"))  NOT (animal OR rat OR mice)) |
| **CINAHL**  **724** | ((multi-domain OR bi-domain OR multidomain OR bidomain OR lifestyle OR multifaceted OR multidisciplinary OR crossdisciplinary OR interdisciplinary OR holistic) AND intervention*)  **AND** (obesity OR "body mass index" OR BMI OR "blood pressure" OR Hypercholesterolemia OR "high cholesterol" OR "cardiovascular disease" OR "Metabolic Syndrome")  **AND** ("Meta-Analysis as Topic" OR ("systematic review" OR "systematic literature review" OR "meta-Analys*" OR "systematic review of the literature" OR "meta-analytical review" OR "systematic review and meta-analys*" OR "systematic review with meta-analys*"))  **NOT** (animal OR rat OR mice) |
| **ProQuest**  **1848** | ((multi-domain OR bi-domain OR multidomain OR bidomain OR lifestyle OR multifaceted OR multidisciplinary OR crossdisciplinary OR interdisciplinary OR holistic) AND intervention*)  **AND** (obesity OR "body mass index" OR BMI OR "blood pressure" OR Hypercholesterolemia OR "high cholesterol" OR "cardiovascular disease" OR "Metabolic Syndrome")  **AND** ("Meta-Analysis as Topic" OR ("systematic review" OR "systematic literature review" OR "meta-Analys*" OR "systematic review of the literature" OR "meta-analytical review" OR "systematic review and meta-analys*" OR "systematic review with meta-analys*"))  **NOT** (animal OR rat OR mice) |
| **PsycInfo**  **237** | ((multi-domain OR bi-domain OR multidomain OR bidomain OR lifestyle OR multifaceted OR multidisciplinary OR crossdisciplinary OR interdisciplinary OR holistic) AND intervention*)  **AND** (obesity OR "body mass index" OR BMI OR "blood pressure" OR Hypercholesterolemia OR "high cholesterol" OR "cardiovascular disease" OR "Metabolic Syndrome")  **AND** ("Meta-Analysis as Topic" OR ("systematic review" OR "systematic literature review" OR "meta-Analys*" OR "systematic review of the literature" OR "meta-analytical review" OR "systematic review and meta-analys*" OR "systematic review with meta-analys*"))  **NOT** (animal OR rat OR mice) |

Table S2. List of the excluded studies.

| **Study** | **Exclusion reason** |
| --- | --- |
| Abbate, M., Gallardo-Alfaro, L., Bibiloni, M. D., & Tur, J. A. (2020). Efficacy of dietary intervention or in combination with exercise on primary prevention of cardiovascular disease: A systematic review. NUTRITION METABOLISM AND CARDIOVASCULAR DISEASES, 30. | Population was “adults at cardiovascular risk”; overweight/obesity was not an explicit inclusion criterion, and ~½ of the 21 studies were diet-only interventions, so the review does not primarily synthesise bi-domain (diet + PA) RCTs in people classified as overweight/obese |
| Ablett, A. D., Boyle, B. R., & Avenell, A. (2019). Fractures in Adults After Weight Loss from Bariatric Surgery and Weight Management Programs for Obesity: Systematic Review and Meta-analysis. Obes Surg, 29. | Focuses on fracture outcomes after weight-loss interventions (bariatric surgery and weight-management programmes), rather than effectiveness of combined diet + physical-activity interventions for obesity/overweight; includes surgical studies and fracture endpoints. |
| Bae, J.-H., & Lee, H. (2021). The effect of diet, exercise, and lifestyle intervention on childhood obesity: A network meta-analysis. CLINICAL NUTRITION, 40. | Network meta-analysis spans diet-only, exercise-only, and mixed ‘lifestyle’ nodes; not restricted to combined diet + physical-activity interventions, so the bidomain effect cannot be isolated consistently. |
| Barr-Anderson, D. J., Adams-Wynn, A. W., DiSantis, K. I., & Kumanyika, S. (2013). Family-focused physical activity, diet and obesity interventions in African-American girls: a systematic review. OBESITY REVIEWS, 14. | Although a systematic review, it targeted prevention and treatment studies in African-American girls regardless of baseline weight; many samples were not confirmed overweight/obese, and several studies were uncontrolled or quasi-experimental, so the review does not principally synthesise RCTs conducted in participants classified as overweight/obese |
| Batrakoulis, A., Jamurtas, A. Z., Metsios, G. S., Perivoliotis, K., Liguori, G., Feito, Y., Riebe, D., Thompson, W. R., Angelopoulos, T. J., Krustrup, P., Mohr, M., Draganidis, D., Poulios, A., & Fatouros, I. G. (2022). Comparative Efficacy of 5 Exercise Types on Cardiometabolic Health in Overweight and Obese Adults: A Systematic Review and Network Meta-Analysis of 81 Randomized Controlled Trials. Circ Cardiovasc Qual Outcomes, 15. | Network meta-analysis of exercise-only RCTs; no dietary component included, so fails the bi-domain criterion |
| Bonvicini, L., Pingani, I., Venturelli, F., Patrignani, N., Bassi, M. C., Broccoli, S., Ferrari, F., Gallelli, T., Panza, C., Vicentini, M., & Rossi, P. G. (2022). Effectiveness of mobile health interventions targeting parents to prevent and treat childhood Obesity: Systematic review. PREVENTIVE MEDICINE REPORTS, 29, Article 101940. | Review of parent-targeted m-health apps; majority of included studies were prevention trials in general-population children (weight status not an eligibility criterion) and several lacked a structured dietary element, so criteria not met |
| Brown, T., & Summerbell, C. (2009). Systematic review of school-based interventions that focus on changing dietary intake and physical activity levels to prevent childhood obesity: an update to the obesity guidance produced by the National Institute for Health and Clinical Excellence. Obes Rev, 10. | School-based prevention review; recruited unselected children and assessed risk of future obesity rather than interventions in already overweight/obese participants |
| Collazo-Castiñeira, P., Sánchez-Izquierdo, M., Reiter, L. J., Bauer, S., Cruz-Jentoft, A. J., Schoufour, J. D., Weijs, P. J. M., & Eglseer, D. (2024). Analysis of behavioral change techniques used in exercise and nutritional interventions targeting adults around retirement age with sarcopenic obesity in a systematic review. Arch Gerontol Geriatr, 123, 105437. | Methodological/secondary analysis of behaviour change techniques (BCTs) in studies from an existing systematic review; not an effectiveness systematic review/meta-analysis of bidomain obesity interventions. |
| Fair, F., & Hora, S. (2021). A meta‐review of systematic reviews of lifestyle interventions for reducing gestational weight gain in women with overweight or obesity. OBESITY REVIEWS, 22. | Meta-review/overview of systematic reviews on gestational weight gain; secondary-level evidence and pregnancy-specific outcome outside this OoSR’s focus on bidomain obesity/overweight interventions in general populations. |
| Franz, M. J., Boucher, J. L., Rutten-Ramos, S., & VanWormer, J. J. (2015). Lifestyle weight-loss intervention outcomes in overweight and obese adults with type 2 diabetes: a systematic review and meta-analysis of randomized clinical trials. J Acad Nutr Diet, 115. | Type 2 diabetes population; interventions largely dietary (including macronutrient-composition comparisons) and not consistently combined with structured physical-activity components—does not primarily synthesise bidomain programmes. |
| Hardeman, W., Griffin, S., Johnston, M., Kinmonth, A. L., & Wareham, N. J. (2000). Interventions to prevent weight gain: a systematic review of psychological models and behaviour change methods. Int J Obes Relat Metab Disord, 24. | Prevention review recruited unselected populations (any baseline weight) and included non-randomised designs; not focused on bi-domain weight-management RCTs in people with overweight/obesity |
| Hasan, B., Nayfeh, T., Alzuabi, M., Wang, Z., Kuchkuntla, A. R., Prokop, L. J., Newman, C. B., Murad, M. H., & Rajjo, T. I. (2020). Weight Loss and Serum Lipids in Overweight and Obese Adults: A Systematic Review and Meta-Analysis. J Clin Endocrinol Metab, 105. | Meta-analysis estimating lipid changes per kg weight loss across lifestyle, pharmacologic and bariatric-surgery interventions; outcome is serum lipids and includes non-lifestyle modalities, not a bidomain diet+PA effectiveness synthesis. |
| Hens, W., Taeyman, J., Cornelis, J., Gielen, J., Van Gaal, L., & Vissers, D. (2016). The Effect of Lifestyle Interventions on Excess Ectopic Fat Deposition Measured by Noninvasive Techniques in Overweight and Obese Adults: A Systematic Review and Meta-Analysis. J Phys Act Health, 13. | Targets ectopic fat deposition outcomes; interventions include exercise or diet (not necessarily combined) and focus is organ fat rather than obesity/overweight management outcomes. |
| Hunter, E., Avenell, A., Maheshwari, A., Stadler, G., & Best, D. (2021). The effectiveness of weight-loss lifestyle interventions for improving fertility in women and men with overweight or obesity and infertility: A systematic review update of evidence from randomized controlled trials. Obes Rev, 22. | Infertility population; primary focus is fertility outcomes in women/men with overweight/obesity rather than general obesity/overweight management outcomes. |
| Kamath, C. C., Vickers, K. S., Ehrlich, A., McGovern, L., Johnson, J., Singhal, V., Paulo, R., Hettinger, A., Erwin, P. J., & Montori, V. M. (2008). Behavioral Interventions to Prevent Childhood Obesity: A Systematic Review and Metaanalyses of Randomized Trials. JOURNAL OF CLINICAL ENDOCRINOLOGY & METABOLISM, 93. | Prevention-focused paediatric review recruited general (not necessarily overweight/obese) children; aim was obesity prevention, not treatment in already overweight/obese participants |
| Katz, D. L., O'Connell, M., Njike, V. Y., Yeh, M. C., & Nawaz, H. (2008). Strategies for the prevention and control of obesity in the school setting: systematic review and meta-analysis. INTERNATIONAL JOURNAL OF OBESITY, 32. | School-based prevention review recruited children 3–18 y regardless of weight status; population therefore not limited to overweight/obese and several single-domain (nutrition-only or PA-only) programmes were pooled |
| King, A., Graham, C. A., Glaister, M., Da Silva Anastacio, V., Pilic, L., & Mavrommatis, Y. (2023). The efficacy of genotype-based dietary or physical activity advice in changing behavior to reduce the risk of cardiovascular disease, type II diabetes mellitus or obesity: a systematic review and meta-analysis. Nutr Rev, 81. | Personalised genotype-based advice trials; interventions delivered either diet or PA advice (not both) and populations were general or “at-risk”, not necessarily overweight/obese |
| Lavelle, M. A., Knopp, M., Gunther, C. W., & Hopkins, L. C. (2023). Youth and Peer Mentor Led Interventions to Improve Biometric-, Nutrition, Physical Activity, and Psychosocial-Related Outcomes in Children and Adolescents: A Systematic Review. Nutrients, 15. | Peer-mentor programmes aimed at obesity prevention; most samples were unselected for weight status and many studies lacked a combined diet + PA component |
| Li, B., Gao, S., Bao, W., & Li, M. (2022). Effectiveness of lifestyle interventions for treatment of overweight/obesity among children in China: A systematic review and meta-analysis. Front Endocrinol. | Review restricted to exercise-only modalities for obese adolescents; no dietary component, thus fails the bi-domain criterion |
| Li, D., & Chen, P. (2021). The Effects of Different Exercise Modalities in the Treatment of Cardiometabolic Risk Factors in Obese Adolescents with Sedentary Behavior—A Systematic Review and Meta-Analysis of Randomized Controlled Trials. Children, 8. | Exercise-only systematic review/meta-analysis in obese adolescents; no dietary component, so not a bidomain intervention. |
| Lutaud, R., Mitilian, E., Forte, J., Gentile, G., Reynaud, R., Truffet, C., & Bellanger, T. (2023). Motivational interviewing for the management of child and adolescent obesity: a systematic literature review. BJGP Open, 7. | Motivational interviewing interventions for child/adolescent obesity; counselling approach is not necessarily a structured combined diet + physical-activity programme (intervention content heterogeneous). |
| Martenstyn, J., King, M., & Rutherford, C. (2020). Impact of weight loss interventions on patient-reported outcomes in overweight and obese adults with type 2 diabetes: a systematic review. J Behav Med, 43. | Type 2 diabetes population; focuses on patient-reported outcomes across various weight-loss interventions (not specifically combined diet+PA). |
| McDowell, K., Petrie, M. C., Raihan, N. A., & Logue, J. (2018). Effects of intentional weight loss in patients with obesity and heart failure: a systematic review. Obes Rev, 19. | Heart failure + obesity population; includes lifestyle, pharmacotherapy and bariatric surgery (and diet-only arms), so not restricted to bidomain interventions in general overweight/obesity cohorts. |
| Monasta, L., Batty, G. D., Macaluso, A., Ronfani, L., Lutje, V., Bavcar, A., van Lenthe, F. J., Brug, J., & Cattaneo, A. (2011). Interventions for the prevention of overweight and obesity in preschool children: a systematic review of randomized controlled trials. Obes Rev, 12. | Population below the ≥ 5 y age threshold; focus is primary prevention in largely normal-weight preschoolers |
| Narzisi, K., & Simons, J. (2021). Interventions that prevent or reduce obesity in children from birth to five years of age: A systematic review. J Child Health Care, 25. | Same age-eligibility issue as above (participants < 5 y) |
| Newton, R. L., Jr., Griffith, D. M., Kearney, W. B., & Bennett, G. G. (2014). A systematic review of weight loss, physical activity and dietary interventions involving African American men. Obes Rev, 15 Suppl 4, 93-106. | Review includes men regardless of baseline weight; many trials recruited normo-weight hypertensive participants and did not focus on bi-domain diet + PA programmes in overweight/obese individuals |
| Obita, G., & Alkhatib, A. (2023). Effectiveness of Lifestyle Nutrition and Physical Activity Interventions for Childhood Obesity and Associated Comorbidities among Children from Minority Ethnic Groups: A Systematic Review and Meta-Analysis. Nutrients, 15. | Review targets prevention in minority-ethnic children and accepts normal-weight participants; population therefore not restricted to individuals already living with overweight/obesity, as required |
| O'Leary, C. B., & Hackney, A. C. (2014). Acute and chronic effects of resistance exercise on the testosterone and cortisol responses in obese males: a systematic review. Physiol Res, 63. | Resistance-exercise–only review; no dietary component, so not a bi-domain intervention |
| Peirson, L., Fitzpatrick-Lewis, D., Morrison, K., Ciliska, D., Kenny, M., Usman Ali, M., & Raina, P. (2015). Prevention of overweight and obesity in children and youth: a systematic review and meta-analysis. CMAJ Open, 3. | Focuses on primary prevention in mixed-weight child/adolescent populations rather than treatment of established overweight/obesity |
| Recchia, F., Leung, C. K., Yu, A. P., Leung, W., Yu, D. J., Fong, D. Y., Montero, D., Chi-Ho, L., Wong, S. H. S., & Siu, P. M. (2023). Dose–response effects of exercise and caloric restriction on visceral adiposity in overweight and obese adults: a systematic review and meta-analysis of randomised controlled trials. BRITISH JOURNAL OF SPORTS MEDICINE, 57. | Compares exercise-only versus caloric-restriction-only RCTs; combined diet + exercise arms intentionally excluded, so not a bi-domain review |
| Rondanelli, M., Gasparri, C., Rigon, C., Ferraris, C., Riva, A., Petrangolini, G., Peroni, G., Faliva, M. A., Naso, M., & Perna, S. (2023). A meta-analysis on the changes of BMI during an inpatient treatment with different follow-up lengths. . | Meta-analysis of inpatient vs. outpatient weight-loss settings; included studies are not limited to RCTs and some programmes rely mainly on diet or psychological support. Fails the “RCTs of bi-domain interventions” criterion |
| Sharkey, T., Whatnall, M. C., Hutchesson, M. J., Haslam, R. L., Bezzina, A., Collins, C. E., & Ashton, L. M. (2020). Effectiveness of gender-targeted versus gender-neutral interventions aimed at improving dietary intake, physical activity and/or overweight/obesity in young adults. . | Targets healthy young adults (17–35 y); many interventions are diet-only or PA-only and participants are not required to have overweight/obesity. |
| Tamin, T. Z., Murdana, N., Pitoyo, Y., & Safitri, E. D. (2018). Exercise Intervention for Chronic Pain Management, Muscle Strengthening, and Functional Score in Obese Patients with Chronic Musculoskeletal Pain: A Systematic Review and Meta-analysis. Acta Med Indones, 50. | Examines exercise vs diet for pain management in obese adults; no intervention arm combines diet and physical activity, so does not meet bi-domain criterion. |
| Tully, L., Arthurs, N., Wyse, C., Browne, S., Case, L., McCrea, L., O'Connell, J. M., O'Gorman, C. S., Smith, S. M., Walsh, A., Ward, F., & O'Malley, G. (2022). Guidelines for treating child and adolescent obesity: A systematic review. Front Nutr, 9, 902865. | Narrative quality appraisal of clinical guidelines; does not synthesise RCTs and lacks interventional data—outside scope. |
| van Dammen, L., Wekker, V., de Rooij, S. R., Groen, H., Hoek, A., & Roseboom, T. J. (2018). A systematic review and meta-analysis of lifestyle interventions in women of reproductive age with overweight or obesity: the effects on symptoms of depression and anxiety. Obes Rev, 19. | Primary outcomes are symptoms of depression/anxiety in women of reproductive age; mental-health outcome focus (not obesity/anthropometric outcomes) and population is pregnancy/reproductive-age specific. |
| van den Hoek, D. J., Miller, C. T., Fraser, S. F., Selig, S. E., & Dixon, J. B. (2017). Does exercise training augment improvements in quality of life induced by energy restriction for obese populations? A systematic review. Qual Life Res, 26. | Compares energy restriction alone vs diet+exercise on health-related quality of life; outcome is HRQOL and includes ER-only comparator, not a bidomain obesity-outcome synthesis. |
| Verheggen, R. J., Maessen, M. F., Green, D. J., Hermus, A. R., Hopman, M. T., & Thijssen, D. H. (2016). A systematic review and meta-analysis on the effects of exercise training versus hypocaloric diet: distinct effects on body weight and visceral adipose tissue. Obes Rev, 17. | Compares exercise-only with hypocaloric-diet-only interventions; it explicitly excludes any arm that combines the two domains, so fails the bi-domain criterion. |
| Verrotti, A., Penta, L., Zenzeri, L., Agostinelli, S., & De Feo, P. (2014). Childhood obesity: prevention and strategies of intervention. A systematic review of school-based interventions in primary schools. A systematic review of school-based interventions in primary schools. | School-based prevention review; participants were general primary-school children (weight status not an inclusion criterion) and many programmes were single-domain. Fails both population and intervention criteria. |
| Webb, E. J., Osmotherly, P. G., & Baines, S. K. (2021). Physical function after dietary weight loss in overweight and obese adults with osteoarthritis: a systematic review and meta-analysis. Public Health Nutr, 24. | Osteoarthritis comorbidity; compares dietary interventions for weight loss on physical function—primarily diet-focused rather than combined diet+physical-activity interventions. |
| Williams, A. J., Henley, W. E., Williams, C. A., Hurst, A. J., Logan, S., & Wyatt, K. M. (2013). Systematic review and meta-analysis of the association between childhood overweight and obesity and primary school diet and physical activity policies. Int J Behav Nutr Phys Act, 10, 101. | Evaluates school diet/PA policies (observational & mixed designs) in general primary-school populations; not limited to overweight/obese children and many policies are single-domain. |
| Yang, L. H., Liang, C., Yu, Y. N., Xiao, Q., Xi, M. M., & Tang, L. X. (2022). Family sports interventions for the treatment of obesity in childhood: a meta-analysis. JOURNAL OF HEALTH POPULATION AND NUTRITION, 41. | Meta-analysis of family-sports RCTs; the vast majority of interventions were exercise/physical-activity only, with dietary elements optional and not analysed separately—does not principally synthesise bi-domain programmes. |
| Zhang, X., Devlin, H. M., Smith, B., Imperatore, G., Thomas, W., Lobelo, F., Ali, M. K., Norris, K., Gruss, S., Bardenheier, B., Cho, P., Garcia de Quevedo, I., Mudaliar, U., Jones, C. D., Durthaler, J. M., Saaddine, J., Geiss, L. S., & Gregg, E. W. (2017). Effect of lifestyle interventions on cardiovascular risk factors among adults without impaired glucose tolerance or diabetes: A systematic review and meta-analysis. PLoS One, 12. | Lifestyle-intervention meta-analysis in adults without impaired glucose tolerance or diabetes; population was not restricted to overweight/obese, and many trials tested single-domain strategies. Fails the population criterion. |

Table S3. Results of methodological quality using AMSTAR2 of included systematic reviews and meta-analysis.

| **Study (Year)** | **1** | **2*** | **3** | **4*** | **5** | **6** | **7*** | **8** | **9*** | **10** | **11*** | **12** | **13*** | **14** | **15*** | **16** | **Overall confidence** |
| --- | --- | --- | --- | --- | --- | --- | --- | --- | --- | --- | --- | --- | --- | --- | --- | --- | --- |
| **Aguilar Cordero et al., 2015** | Y | N | Y | PY | N | N | N | Y | N | N | No MA | N | N | Y | No MA | PY | Critically low |
| **Albornoz-Guerrero et al., 2021** | Y | N | Y | PY | N | N | N | Y | Y | N | No MA | N | PY | PY | No MA | Y | Critically low |
| **AlMarzooqi et al., 2011** | PY | N | N | N | N | N | N | PY | N | N | No MA | N | N | N | No MA | N | Critically low |
| **Al-Mhanna et al., 2023** | Y | Y | Y | Y | Y | Y | Y | Y | Y | N | Y | Y | Y | Y | Y | Y | High |
| **Angawi & Gaissi, 2021** | Y | N | PY | Y | Y | Y | N | Y | N | N | No MA | N | N | No MA | No MA | Y | Critically low |
| **Appuhamy et al., 2014** | Y | N | Y | Y | PY | PY | N | Y | Y | N | Y | PY | PY | Y | Y | Y | Low |
| **Baillot et al., 2015** | Y | N | Y | Y | Y | PY | N | Y | Y | N | Y | PY | PY | Y | N | Y | Low |
| **Barte et al., 2014** | Y | N | Y | PY | Y | PY | N | Y | N | N | Y | N | N | Y | Y | Y | Critically low |
| **Batsis et al., 2017** | Y | N | Y | PY | PY | N | N | Y | N | N | No MA | N | N | PY | No MA | Y | Critically low |
| **Best et al., 2017** | Y | Y | Y | Y | PY | Y | N | Y | Y | N | Y | PY | PY | Y | PY | Y | Low |
| **Bondyra-Wiśniewska et al., 2021** | Y | N | PY | PY | N | N | N | Y | N | N | N | N | N | PY | N | Y | Critically low |
| **Brown et al., 2015** | Y | N | Y | Y | PY | PY | N | Y | N | N | Y | N | N | Y | Y | Y | Critically low |
| **Gea Cabrera et al., 2021** | Y | N | Y | Y | Y | Y | N | Y | N | N | Y | N | N | Y | Y | Y | Critically low |
| **Hassan et al., 2016** | Y | N | Y | Y | Y | Y | N | Y | Y | N | No MA | PY | PY | No MA | No MA | Y | Low |
| **Johns et al., 2014** | Y | N | Y | Y | Y | Y | N | Y | Y | N | Y | PY | PY | Y | Y | Y | Low |
| **Khalafi et al., 2023a** | Y | N | Y | Y | Y | Y | Y | Y | Y | N | Y | Y | Y | Y | Y | Y | Moderate |
| **Khalafi et al., 2023b** | Y | Y | Y | Y | Y | Y | Y | Y | Y | N | Y | Y | Y | Y | Y | Y | High |
| **Liang et al., 2022** | Y | Y | Y | Y | Y | Y | Y | Y | Y | N | Y | Y | Y | Y | Y | Y | High |
| **Ling et al., 2016** | Y | N | Y | PY | N | N | N | Y | N | N | No MA | N | N | No MA | No MA | N | Critically low |
| **Liu et al., 2021** | Y | N | Y | Y | Y | Y | Y | Y | Y | N | Y | Y | Y | Y | Y | Y | High |
| **Mattos et al., 2022** | Y | Y | Y | Y | Y | Y | N | Y | Y | N | No MA | PY | PY | No MA | No MA | Y | Low |
| **McGovern et al., 2008** | Y | Y | Y | Y | Y | Y | N | Y | Y | N | Y | Y | Y | Y | Y | Y | High |
| **Merlotti et al., 2014** | Y | N | Y | Y | Y | Y | N | Y | N | N | Y | N | N | Y | Y | Y | Critically low |
| **Miller et al., 2013** | Y | N | Y | Y | Y | Y | N | Y | PY | N | No MA | PY | PY | No MA | No MA | Y | Low |
| **Olateju et al., 2023** | Y | Y | Y | PY | Y | Y | Y | Y | N | N | Y | N | N | N | N | Y | Low |
| **Peirson et al., 2015** | Y | Y | Y | Y | Y | Y | Y | Y | Y | N | Y | Y | Y | Y | Y | Y | High |
| **Perez et al., 2019** | Y | N | Y | PY | Y | Y | N | Y | N | N | Y | PY | N | Y | N | Y | Critically low |
| **Rotunda et al., 2024** | Y | N | Y | Y | Y | PY | N | Y | N | N | Y | PY | N | Y | N | Y | Critically low |
| **Ruiz-Gonzalez et al., 2024** | Y | Y | Y | Y | Y | Y | Y | Y | Y | Y | Y | Y | Y | Y | Y | Y | High |
| **Salam et al., 2020** | Y | N | Y | Y | Y | Y | N | Y | Y | N | Y | Y | Y | Y | Y | Y | Moderate |
| **Schwingshackl et al., 2014** | Y | Y | Y | Y | Y | Y | N | Y | Y | N | Y | Y | Y | Y | Y | Y | High |
| **Selvendran et al., 2018** | Y | N | Y | Y | Y | Y | N | Y | Y | N | Y | Y | Y | Y | Y | Y | Low |

**AMSTAR 2 =** A MeaSurement Tool to Assess Reviews tool 2; **Y** = Yes; **N** = No; **PY** = Partially yes; **No MA** = Not meta-analysis; **1 =** Word research question and inclusion criteria according to PICOS (population, intervention, comparison, outcome, study design); **2 =** Establish methods prior to the conduct of the meta-analyses (written protocol); **3 =** Explain the choice of study design for inclusion; **4 =** Use comprehensive literature search strategy; **5 =** Perform study selection in duplicate; **6 =** Perform data extraction in duplicate; **7 =** Provide a list of excluded studies to justify the exclusion; **8 =** Describe the included studies in detail; **9 =** Assess the risk of bias; **10 =** Reported sources of funding for included studies; **11 =** Use appropriate method for statistical combination of results; **12 =** Assess the potential impact of risk of bias for included studies; **13 =** Account for risk of bias while interpreting/discussing the results; **14 =** Explain/discuss any heterogeneity; **15 =** Assess publication bias and discuss its impact on the results; **16 =** Report potential sources of conflict of interest and describe any funding; *** =** Critical weaknesses.

Table S4. Overlap of included studies

| Outcome | c | N | r | CCA % | Interpretation |
| --- | --- | --- | --- | --- | --- |
| BMI, Body weight | 29 | 719 | 647 | 0.4 | Slight overlap |
| Waist circumference | 15 | 147 | 143 | 0.2 | Slight overlap |
| Body composition | 13 | 144 | 137 | 0.4 | Slight overlap |
| Metabolic markers | 16 | 193 | 176 | 0.6 | Slight overlap |
| Blood pressure | 10 | 84 | 82 | 0.3 | Slight overlap |
| Quality of life | 5 | 11 | 11 | 0.0 | No detected overlap |
| Physical function | 9 | 69 | 65 | 0.8 | Slight overlap |
| Dietary behavior | 2 | 8 | 8 | 0.0 | No detected overlap |
| Inflammatory markers | 2 | 21 | 21 | 0.0 | No detected overlap |
| Prevalence | 2 | 12 | 12 | 0.0 | No detected overlap |

CCA: corrected covered area; N: number of included publications; r: number of index publications; c: number of reviews.

## Supplementary Figures


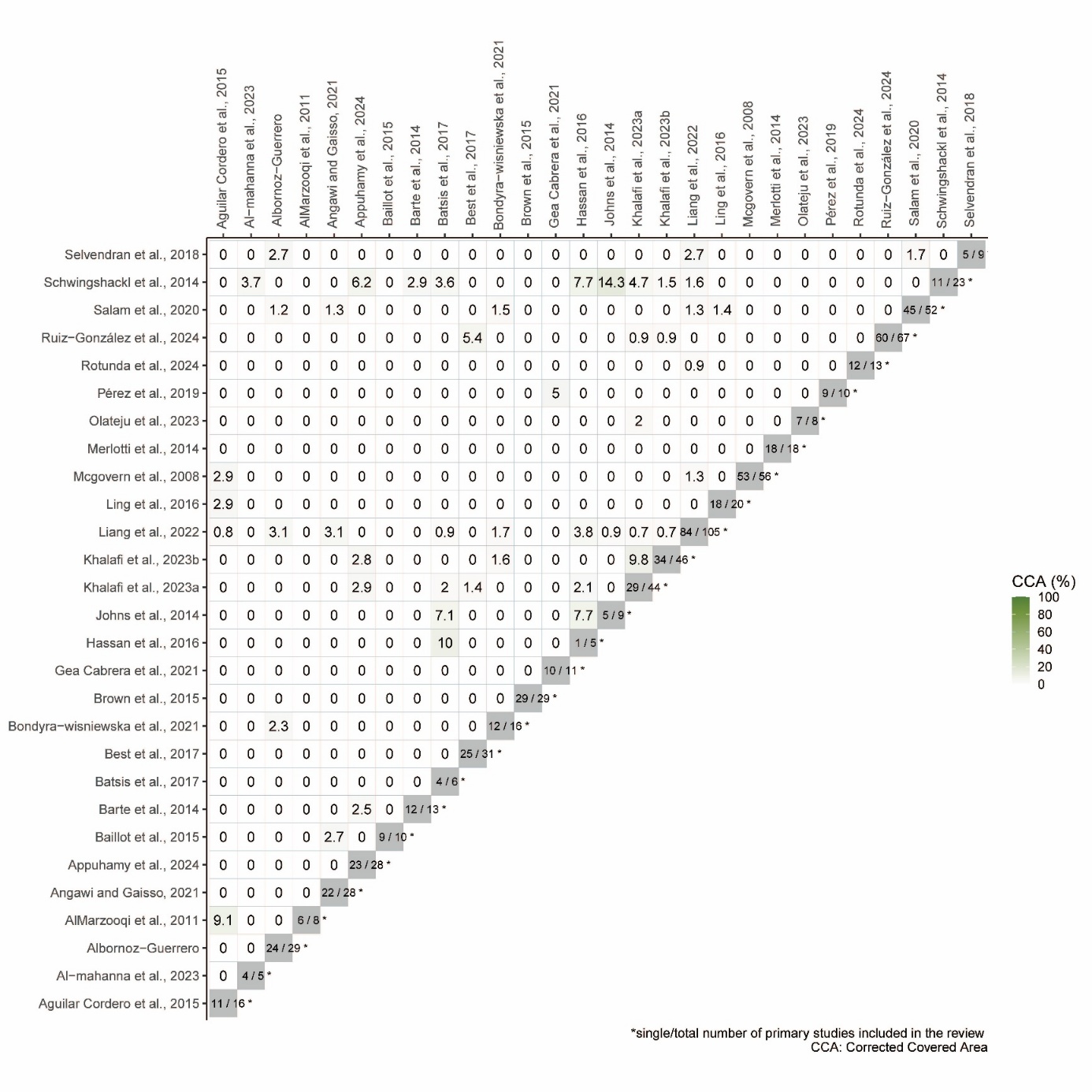


Figure S1. Heatmap for visualization of the degree of overlap of primary studies between pairs for Body weight/BMI .

Note. The degree of overlap of primary studies between pairs of reviews (CCA=0% represents no overlap of primary studies [white colour], CCA=100% represents complete overlap of primary studies between the SRs [deep green colour]). The grey diagonal tiles present the single/ total number of primary studies that were included in each review.


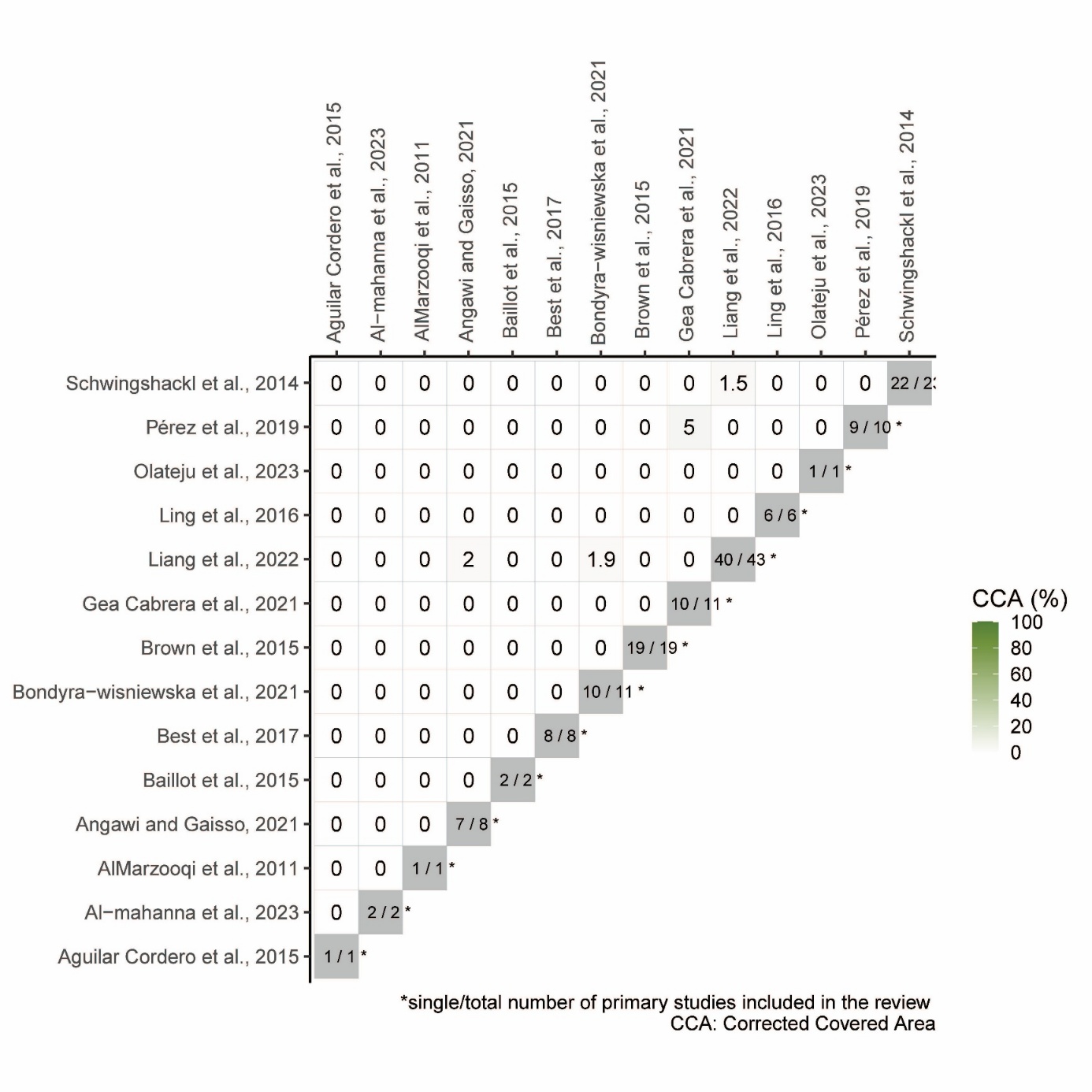


Figure S2. Heatmap for visualization of the degree of overlap of primary studies between pairs for waist circumference.

Note. The degree of overlap of primary studies between pairs of reviews (CCA=0% represents no overlap of primary studies [white colour], CCA=100% represents complete overlap of primary studies between the SRs [deep green colour]). The grey diagonal tiles present the single/ total number of primary studies that were included in each review.


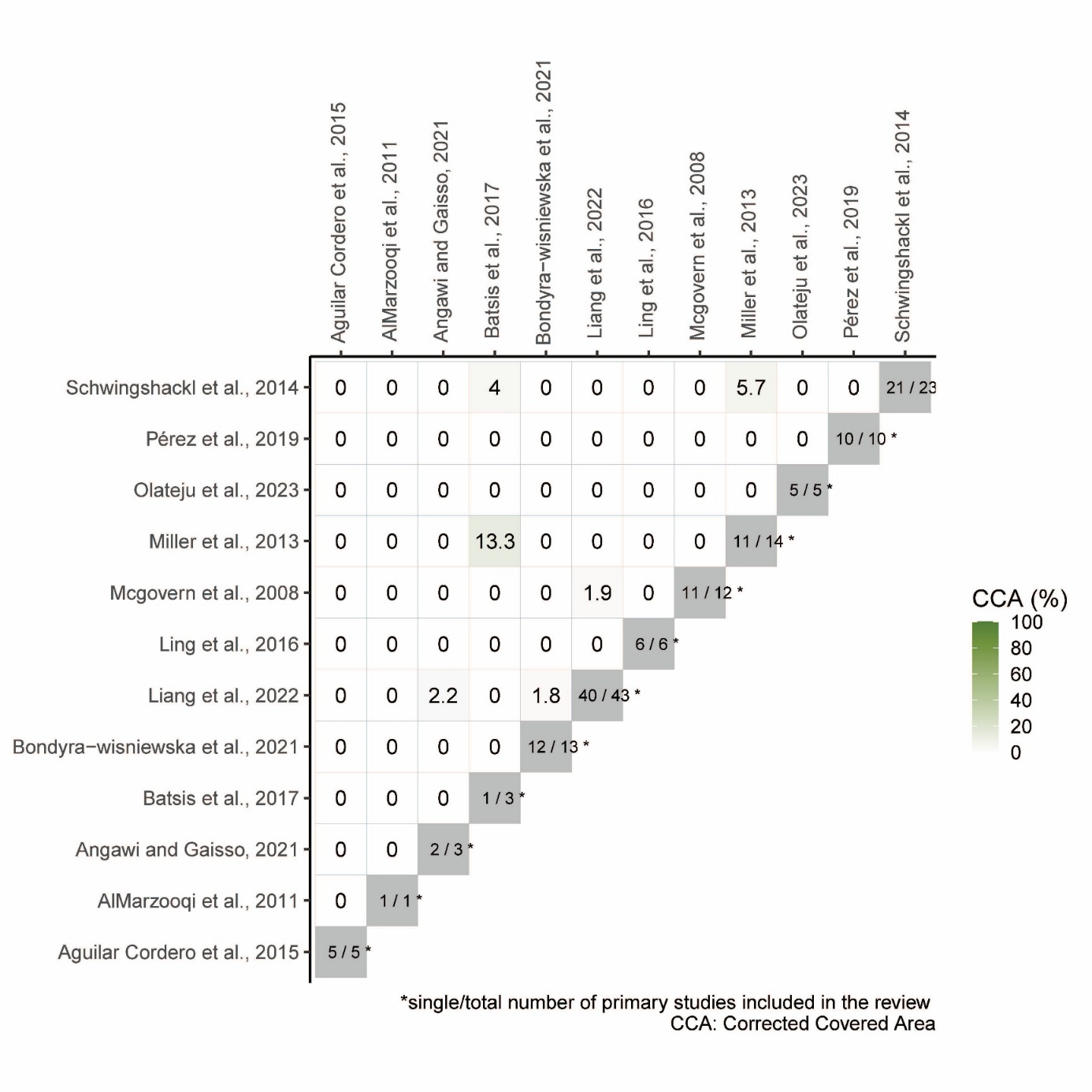


Figure S3. Heatmap for visualization of the degree of overlap of primary studies between pairs for body composition.

Note. The degree of overlap of primary studies between pairs of reviews (CCA=0% represents no overlap of primary studies [white colour], CCA=100% represents complete overlap of primary studies between the SRs [deep green colour]). The grey diagonal tiles present the single/ total number of primary studies that were included in each review.


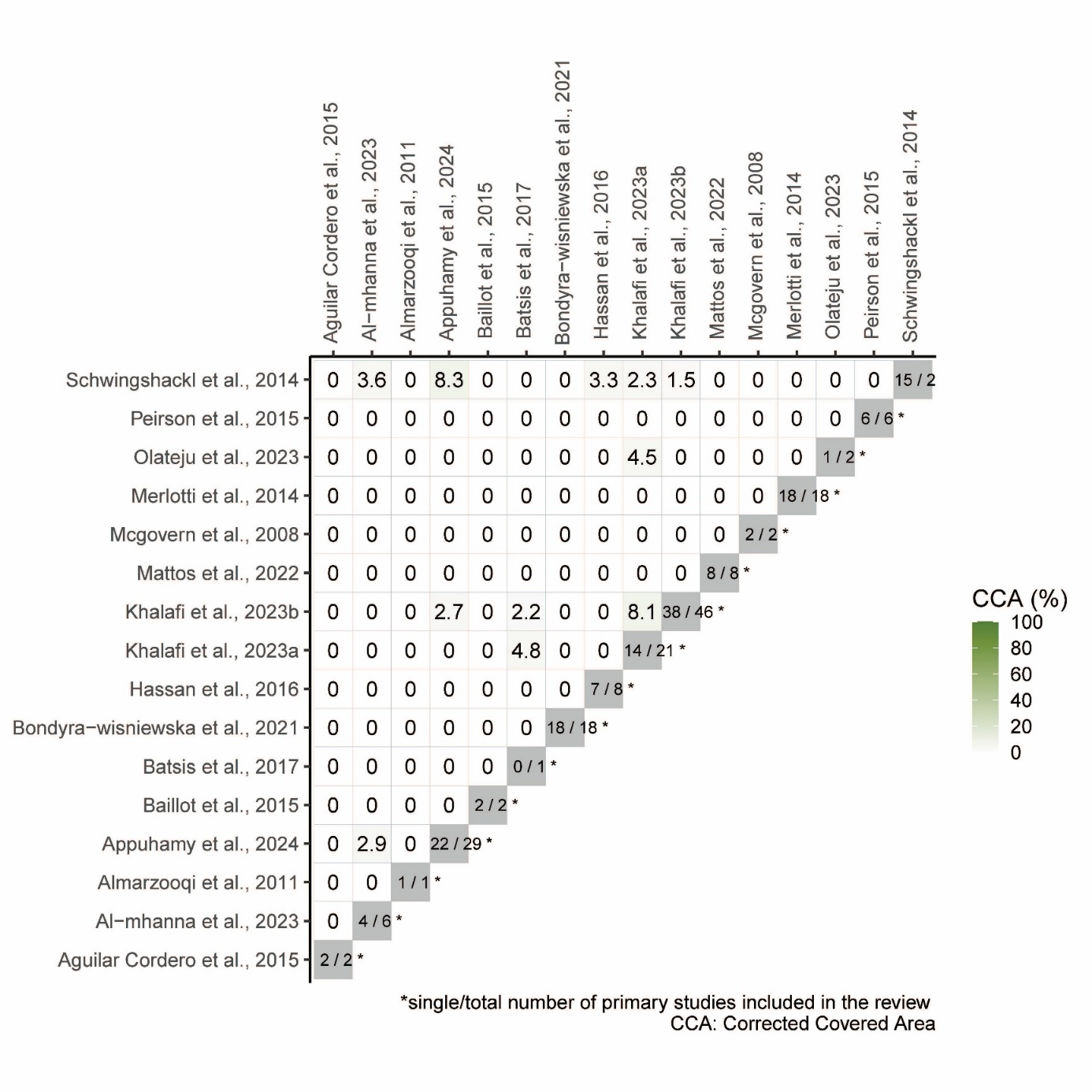


Figure S4. Heatmap for visualization of the degree of overlap of primary studies between pairs for Metabolic markers.

Note. The degree of overlap of primary studies between pairs of reviews (CCA=0% represents no overlap of primary studies [white colour], CCA=100% represents complete overlap of primary studies between the SRs [deep green colour]). The grey diagonal tiles present the single/ total number of primary studies that were included in each review.


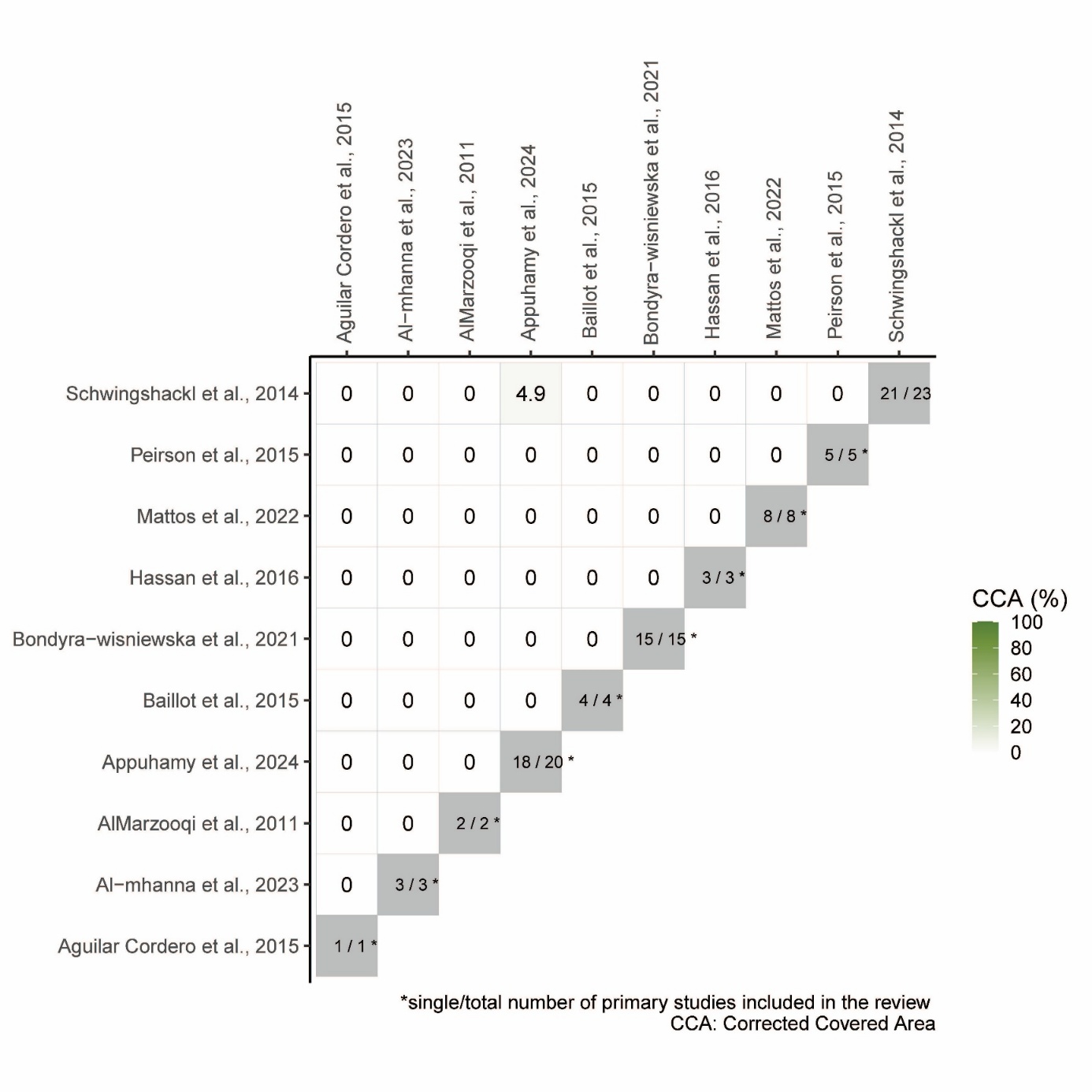


Figure S5. Heatmap for visualization of the degree of overlap of primary studies between pairs Blood pressure.

Note. The degree of overlap of primary studies between pairs of reviews (CCA=0% represents no overlap of primary studies [white colour], CCA=100% represents complete overlap of primary studies between the SRs [deep green colour]). The grey diagonal tiles present the single/ total number of primary studies that were included in each review.


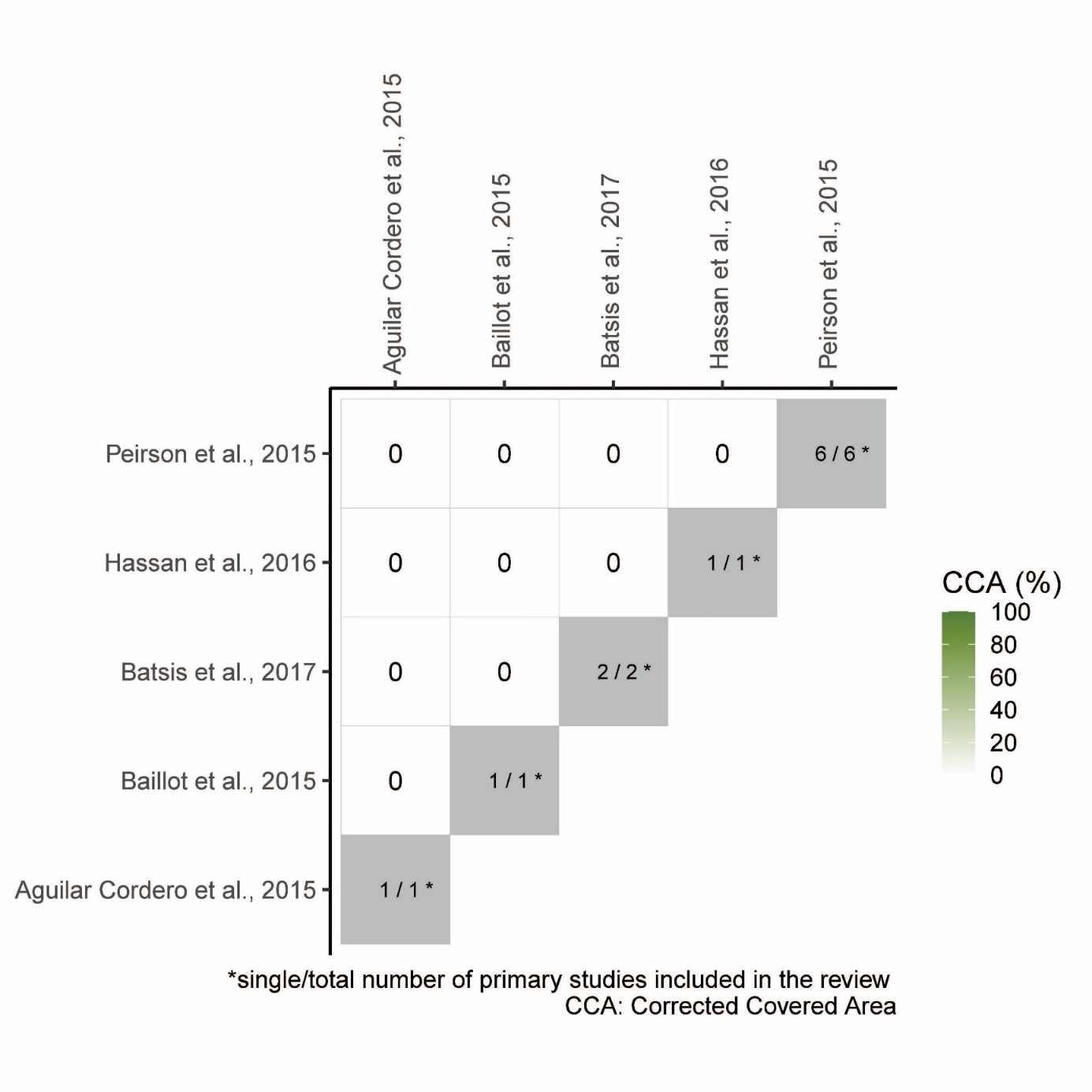


Figure S6. Heatmap for visualization of the degree of overlap of primary studies between pairs for Quality of life.

Note. The degree of overlap of primary studies between pairs of reviews (CCA=0% represents no overlap of primary studies [white colour], CCA=100% represents complete overlap of primary studies between the SRs [deep green colour]). The grey diagonal tiles present the single/ total number of primary studies that were included in each review.


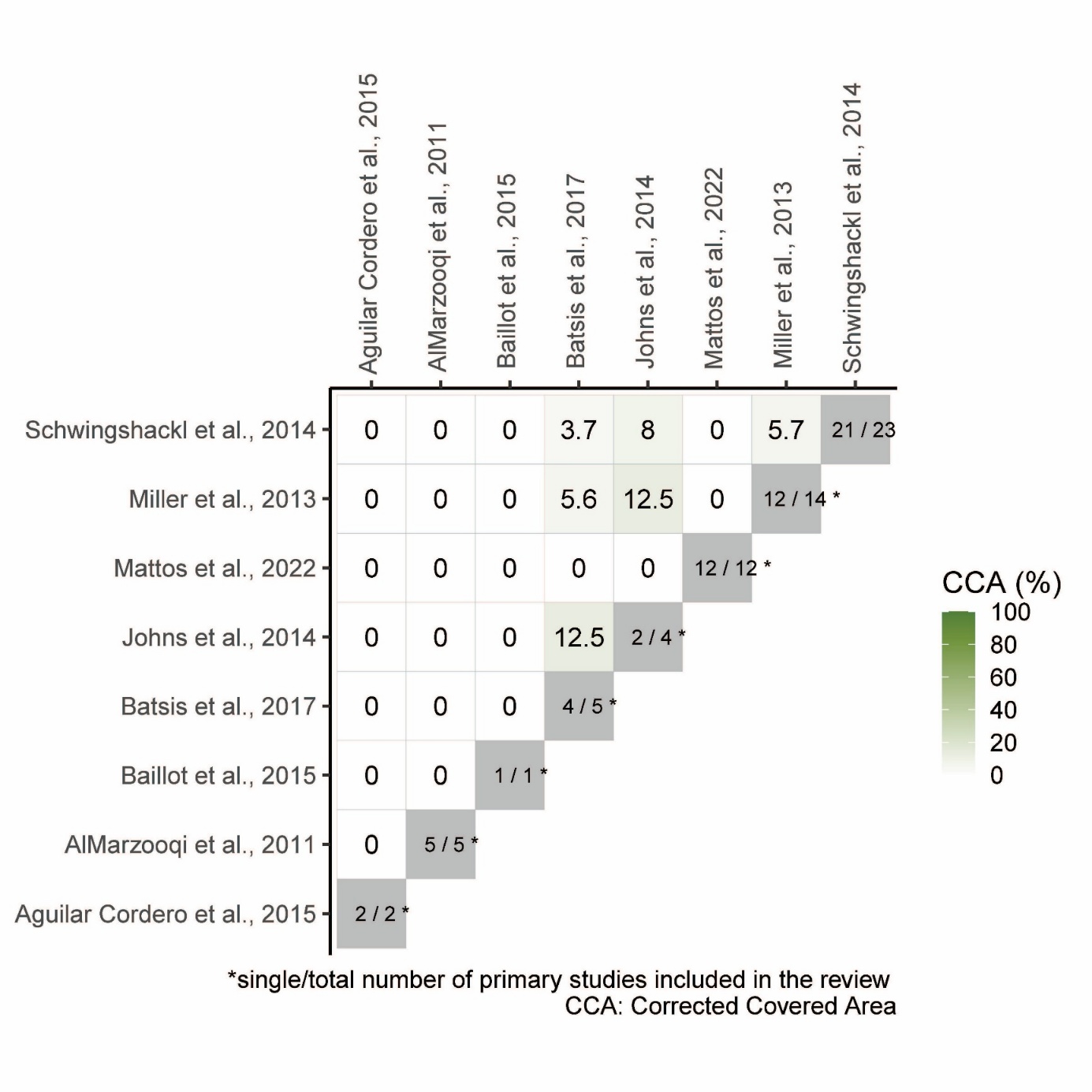


Figure S7. Heatmap for visualization of the degree of overlap of primary studies between pairs for physical function.

Note. The degree of overlap of primary studies between pairs of reviews (CCA=0% represents no overlap of primary studies [white colour], CCA=100% represents complete overlap of primary studies between the SRs [deep green colour]). The grey diagonal tiles present the single/ total number of primary studies that were included in each review.


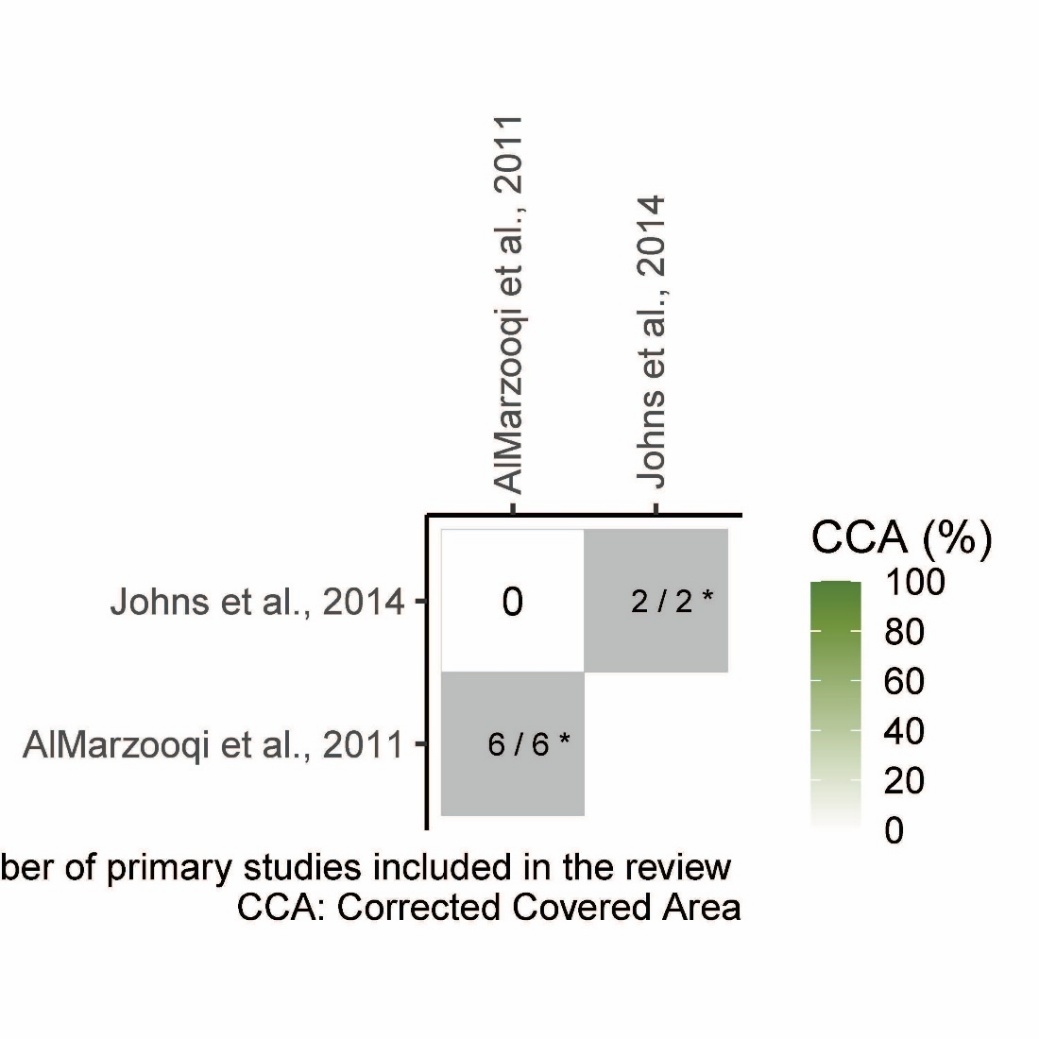


Figure S8. Heatmap for visualization of the degree of overlap of primary studies between pairs for dietary behavior.

Note. The degree of overlap of primary studies between pairs of reviews (CCA=0% represents no overlap of primary studies [white colour], CCA=100% represents complete overlap of primary studies between the SRs [deep green colour]). The grey diagonal tiles present the single/ total number of primary studies that were included in each review.


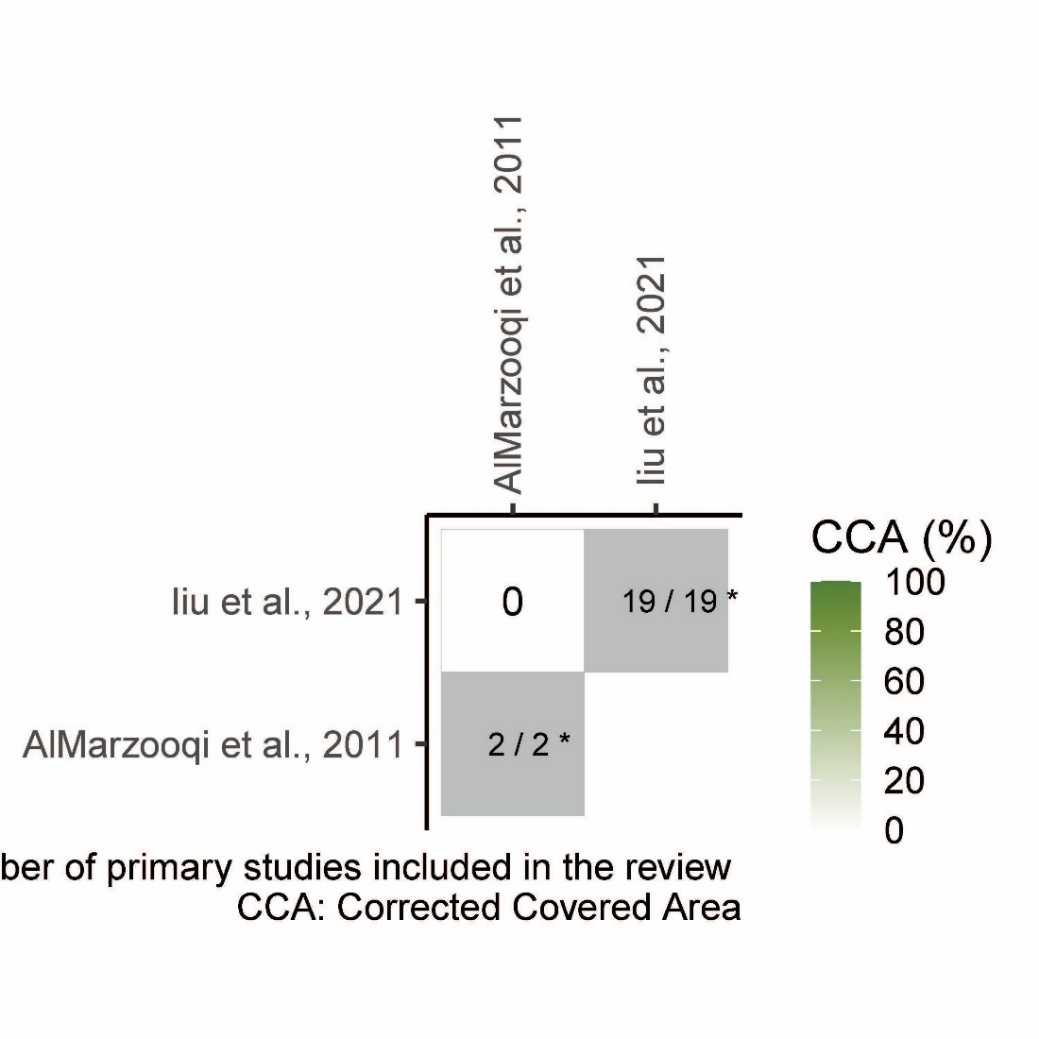


Figure S9. Heatmap for visualization of the degree of overlap of primary studies between pairs for inflammatory markers.

Note. The degree of overlap of primary studies between pairs of reviews (CCA=0% represents no overlap of primary studies [white colour], CCA=100% represents complete overlap of primary studies between the SRs [deep green colour]). The grey diagonal tiles present the single/ total number of primary studies that were included in each review.


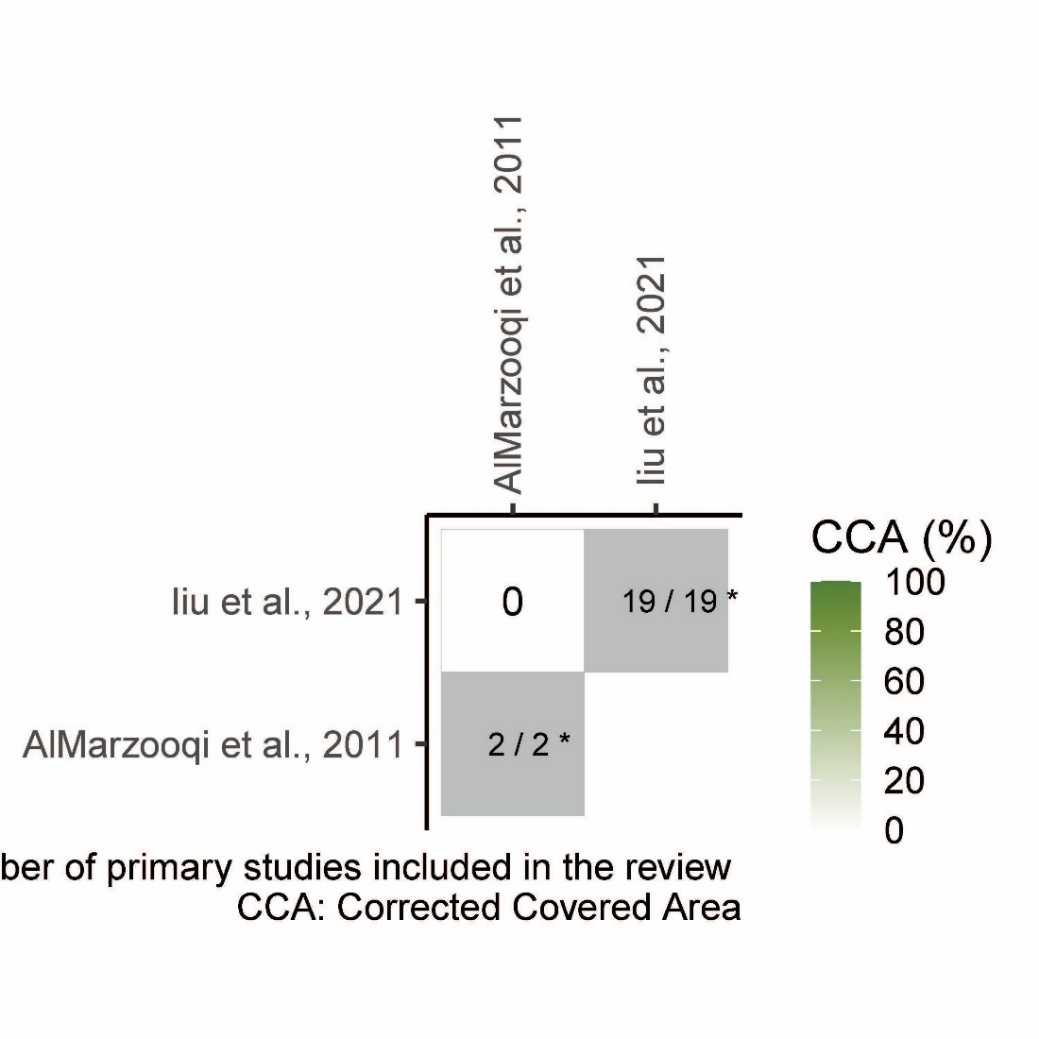


Figure S10. Heatmap for visualization of the degree of overlap of primary studies between pairs for prevalence.

Note. The degree of overlap of primary studies between pairs of reviews (CCA=0% represents no overlap of primary studies [white colour], CCA=100% represents complete overlap of primary studies between the SRs [deep green colour]). The grey diagonal tiles present the single/ total number of primary studies that were included in each review.
